# Association between the different basic activities of daily living on the Barthel index and community living, use of mobility aids, and the survival at 5 years

**DOI:** 10.3389/fpubh.2026.1825340

**Published:** 2026-06-04

**Authors:** Vicente Martín Moreno, Elena Pérez Rico, Amanda Martín Fernández, Jorge Hurtado Gallar, Irene Sánchez González, Elena Sánchez Rodríguez, María Inmaculada Martínez Sanz, Miriam Fernández Gallardo, Juana Marcos Guerra, María del Carmen Cubero Sobrados, Laura Flórez Busto, Asunción García Mateos, Beatriz Alonso Serna, Annly Luzmila Vásquez Alay

**Affiliations:** 1 Orcasitas Health Care Center, Research Institute of the Doce de Octubre Hospital, Madrid, Spain; 2 Orcasitas Health Care Center, Madrid, Spain; 3 Polibea Concert, Madrid, Spain; 4 UNAD Group Manager, Orcasitas Health Care Center, Madrid, Spain

**Keywords:** Barthel index, basic activities of daily living, dependence, functional impairment, instrumental activities of daily living, mobility

## Abstract

**Introduction:**

The Barthel index has designed to assess functional capacity to perform basic activities of daily living. This study analyzes the association between each of these activities and community living, the use of mobility aids, and various socioeconomic factors, as well as survival associated with each activity at 5 years.

**Methods:**

Study conducted between 2020 and 2025 in the functionally dependent population (Barthel ≤60) of the Orcasitas neighborhood in Madrid (Spain). A total of 127 people participated, with a mean age of 86 ± 6.3 years. Barthel index activities were classified into three groups, and the relationship between community living and the use of mobility aids with the level of dependency and survival was analyzed.

**Results:**

One in four people in this cohort was dependent for activities included in group 2 (dressing-undressing, grooming, bathing), but only one in ten was dependent for activities in group 1 (walking independently, chair-to-bed transfers, going up-down stairs, and toilet use) and group 3 (feeding or maintaining bladder and bowel control). Dependence on others on feeding, toilet use, chair-to-bed transfers, and bathing, as well as fecal incontinence, were associated with the need to live with other people. Chair-to-bed transfers, up-down stairs, and urinary incontinence were associated with living homebound. Feeding, toileting, bathing, dressing-undressing, and urinary or fecal incontinence were associated with the need for a hired caregiver. Only bathing was not associated with the use of mobility aids. The activities that showed the strongest association with mortality risk were using the toilet, dressing-undressing, and chair-to-bed transfers.

**Conclusion:**

Dependence on others to perform each of the activities on the Barthel index follows a different pattern with respect to the interrelationships established with dependence on other activities and the personal and social adaptations that this dependence imposes. These characteristics must take account when designing population studies and planning social and health care services.

## Introduction

The assessment of functional capacity is an essential part of the evaluation of older adults, and the Barthel index is usually used for this purpose (1). This tool was designed by consensus among experts, who also determined the weighting in the form of a score assigned to each activity evaluated within this index. Despite the time that has elapsed since its publication, there have been few studies evaluating the relative weight of each of these activities within the index in relation to global functional capacity (2–4). Less frequently has addressed how isolated dependence in each of these activities affected the personal or social sphere of the person with functional dependence, or their survival (5–7).

*A priori* it seems clear that this impact does not necessarily have to be the same for all activities in the Barthel index (8, 9). In fact, the empirical impression is that it should be different (10). Among the activities included in the Barthel index, the original model gave priority to two activities, whose independence received a higher score (1). These two activities, chair-to-bed transfers and walking independently, have a direct association with mobility.

Mobility is a relevant factor. Loss of mobility has been associated with increased frailty and a higher risk of mortality (10–12). This loss of mobility could affect the ability to perform each of the basic activities of daily living included in the Barthel index in diverse ways. Therefore, it seemed important to assess the extent to which this occurred.

In this context, we have come to accept that aging is synonymous with a decline in physical abilities, and that the gradual difficulty in performing simple daily routines, such dressing-undressing, bathing, or grooming, is part of this process. This normalization leads to a certain degree of invisibility, causing the transition from frailty to functional disability among older adults to often go undetected until functional limitations are already clearly evident.

However, even small changes in the ability to dress and undress, or in the ability to bathe or groom without assistance, can serve as early warning signs of frailty (13) that may indicate the onset of muscle weakness. Muscle mass loss, associated with age, a sedentary lifestyle, or other factors, is a progressive process that is not always accompanied by weight loss but does lead to a loss of strength. At the same time, muscle weakness and loss of strength ultimately affect balance. And balance is a key factor in the development of functional dependence (14, 15).

In the early stages, these three factors may manifest as difficulties performing basic movements, such as raising the arms or legs to dress, undress, or groom oneself, or to get into the bathtub or shower. All of this occurs without the mobility reflected in the ability to walk being affected yet (15).

The mobility domain of intrinsic capacity mainly assesses the ability to walk. However, in a society where population aging is a reality, it would be necessary to broaden this criterion, particularly when evaluating this capacity in individuals with functional dependence. In this population group, the Barthel index could play a significant role (11).

The differentiation of Barthel index activities into those associated with mobility (walking, chair-to-bed transfers, going up and down stairs) and those not associated with mobility (the rest) has already been proposed in other studies (11, 16). However, it is likely that the activity of toileting should also be included among the activities associated with mobility. Regarding the remaining activities, it has been suggested that dressing-undressing, grooming, and bathing might describe a new group (17), in which balance and mobility are necessary for their performance (15). In some studies, these three activities are the ones that most frequently require assistance from others (13), and their prevalence could suggest an early onset in the progression of dependency (17), while in others they are relevant factors in the institutionalization of older adults (18).

These criteria would complement the assessment carried out using Fried’s frailty phenotype (17), or any other frailty index (19), by including factors that are commonly considered normal but could serve as early warning signs, whose accumulation increases vulnerability and mortality (19). It would also allow for the identification of population subgroups susceptible to interventions aimed at promoting healthy aging and reducing vulnerability (17, 20).

On the other hand, this functional assessment should also consider the impact of dependency on each activity within the personal and social environment (21–23). This environment could potentially assess how this dependency limited the ability to leave the home, promoted living homebound, was associated with the need to live with other people, or required the use of mobility aids to try to maintain personal and social independence as far as possible (24–26).

Finally, dependency often requires assistance from support people who help with household tasks or the personal care of dependent people (27–29). Therefore, it would be relevant to know how dependency in each activity of the Barthel index influenced the need for these support measures.

Taking these premises into account and noting that the topic has not sufficiently addressed in specific research, we decided to conduct this study, whose objectives were: (1) To analyze the association between each Barthel index activity and living arrangements in the community. (2) To analyze the association between each Barthel index activity and the use of assistive mobility devices. (3) To analyze the association between each Barthel index activity and the availability of assistants for domestic tasks and personal care, as well as live-in caregivers. (4) To analyze the association of each Barthel index activity with various socioeconomic factors. (5) To analyze the association of each Barthel index activity with survival at 5 years of follow-up.

## Materials and methods

### Study design and study population

A prospective observational cohort study was designed with a five-year longitudinal follow-up period, conducted between June 2020 and June 2025 at the Orcasitas health center in Madrid, Spain. This study included the entire population aged sixty-five or older registered at this health center who were functionally dependent on basic activities of daily living (ADL). This data obtained from the e-SOAP application registry, which includes 150 patients. Exclusion criteria were not at the usual place of residence during the study period (*n* = 9), absence of Barthel score recorded in the last year (*n* = 5), and patient refusal to participate in the study (*n* = 5). Between obtaining the data from the registry and conducting the study, four patients died. Finally, A total of 127 patients participated. To establish the criterion of functional dependency, the cutoff point established, according to the Protocol for Care of Dependent Persons of the Community of Madrid, was to have a Barthel index score ≤60.

### Data collection and definition of variables

Information on social factors has collected through an anonymous survey administered by nurses trained for this purpose. Data on economic status was obtained using the Individual Health Card application, which establishes two categories: 1: income above 11,200 euros/year; and 2: income below 11,200 euros/year. This cutoff point has assigned by the Madrid Regional Health Department as a criterion for co-payment in pharmacy services, obtaining free services when income is less than 11,200 euros/year. Therefore, having an income of less than 11,200 euros/year is equivalent to having a low income.

Functional capacity for performing basic activities of daily living has assessed using the Barthel index. Each activity is recorded individually. Each activity has then dichotomized, using the criterion that being dependent or needing significant help to perform the activity was equivalent to being dependent, and being independent or needing minimal help for the activity was equivalent to being independent.

Mobility is a fundamental factor in performing activities, including those included in the Barthel index. Based on clinical experience and previous studies on the hierarchical nature of functional decline (13, 30, 31), we operationally classified the 10 Barthel index activities into three groups according to the level of mobility and postural balance required for their execution: (1) activities where mobility is a relevant factor that conditions their performance: chair-to-bed transfers, going up and down stairs, walking independently, and toilet use. (2) activities where mobility is necessary but do not totally condition their performance: dressing-undressing, grooming, and bathing. (3) Activities where mobility is not an essential factor for their execution or is unnecessary: feeding, urinary incontinence, fecal incontinence (Table 1). This classification is conceptual and pragmatic; its face validity is partially supported by the differential outcomes observed in this study across the three groups.

**Table 1:** Operational classification of activities included in the Barthel index in relation to the involvement of the mobility factor in their execution.

Mobility and Barthel index	
Group Barthel index	Activities
Group 1: Activities where mobility is a relevant factor that conditions their performance	Chair-to-bed transfers Going up and down stairs Walking independently Toilet use
Group 2: Activities where mobility is necessary but do not totally condition their performance, and the postural balance has a role	Dressing-undressing Grooming Bathing
Group 3: Activities where mobility is not an essential factor for their execution or is not required	Feeding Urinary incontinence Fecal incontinence

Cell color format: Mustard color: group 1 activities of the Barthel index. Green color: group 2 activities of the Barthel index. Blue color: group 3 activities of the Barthel index.

Regarding the other variables analyzed, the criteria used were: (1) Educational level, evaluated in two categories: having an education and not having an education. No education included being illiterate; knowing how to read and write but having no education; and not having completed primary education. (2) Living arrangements in the community, classified as living independently (alone or with a partner), living with children, and living with people other than a partner or children. (3) Housing situation in the community, classified as being able to leave the house or being homebound. Within the ability to leave the home, the analysis considered whether this activity occurred independently or accompanied by other people. (4) As instrumental activities, shopping ability was analyzed, classifying people according to whether they could shop independently, with the help of another person, or were unable to make the purchases necessary for daily sustenance. (5) Analysis also included the influence of the need to use mobility aids: crutches, walking sticks, walkers, or wheelchairs.

Finally, the date of death of the participants in this study was obtained through the Madrid Health Service computer system, analyzing overall mortality at 5 years. The professional who conducted this follow-up did not have access to the participants’ medical records or the data obtained in the surveys. Similarly, the healthcare professionals participating in this study did not have access to the computer systems where the date of death was recorded.

### Data analysis

Data analysis was conducted using the SPSS® 18.0 statistical software package. At first, the sample was described by calculating the mean and standard deviation for continuous variables, and by reporting numbers and proportions for categorical variables. Continuous variables were compared using either Student’s *t*-test or the Mann–Whitney U test, while categorical variables were examined with the Chi-square test. The odds ratio (OR) was used to assess the likelihood of an event taking place.

A univariate analysis was conducted to determine which factors were linked to the long-term survival of those in this group. Covariates found to be significant in the bivariate analysis were incorporated into the survival analysis through Cox regression. A proportional hazards survival regression model was applied using Cox regression. The model was described by its estimated coefficients, *p*-values, and Hazard Ratios, each accompanied by their 95% confidence intervals. Linear regression analysis using a stepwise approach was conducted as post-hoc testing. Finally, the areas under the ROC curves were obtained to evaluate the discriminatory capacity with respect to survival for each Barthel index activity. This analysis was performed for each group in the operational classification described, compared with that obtained for the Barthel index. The areas under the ROC curve were compared using the DeLong method, with the Epidat 3.1 statistical package. In this study, any *p* < 0.05 was considered significant.

## Results

The sociodemographic characteristics of this cohort showed a population profile of people with no education (88.1%) of advanced age (86 ± 6.304), who were mostly women (78.7%), and where half of its members (47.2%) had low incomes. In this cohort, being widowed (64.6%) was the most usual marital status, and living with children (63%, *n* = 80) was the most usual form of living arrangements in the community (Table 2).

**Table 2:** Sociodemographic and socioeconomic data of the functionally dependent population of Orcasitas (Orcasitas cohort).

Sociodemographic and socioeconomic data of the functionally dependent population of Orcasitas cohort	
Variable	Value
Age (mean ± SD)	86 ± 6.304
BMI	28.58 ± 4.701
Sex	
Male	21.3% (27)
Female	78.7% (100)
Barthel index level	
Severe (under 40)	29.9% (38)
Moderate (40–60)	70.1% (89)
Marital status	
Married	30.7% (39)
Widower	64.6% (82)
Others	4.7% (6)
Live with a partner	
Yes	30.7% (39)
No	67.7% (86)
Home task assistant—personal caregiver	
Yes	72.4% (92)
No	27.6% (35)
Public assistant at home	
Yes	55.1% (70)
No	44.1% (56)
No answer	0.8% (1)
Private home assistant	
Yes	35.4% (45)
No	62.2% (79)
No answer	2.4% (3)
Internal caregiver	
Yes	18.9% (24)
No	79.5% (101)
No answer	1.6% (2)
Income level	
Less income (<11,200 euros/year)	47.2% (60)
Non low income (≥11,200 euros/year)	52.8% (67)
Education	
Insufficient	88.1% (111)
Had education	11.9% (15)
Housing situation	
Leaves home	64.6% (82)
Homebound	35.4% (45)
Community living arrangements	
Lives independently	37% (47)
Lives with children	44.9% (57)
Lives with others who are not children	18.1% (23)

Being dependent or independent in performing any of the activities on the Barthel index was not associated in this cohort with gender or educational level. Regarding income level, the only association observed was between a higher income level and a greater probability of being able to walk independently (OR 2.548; CI 1.037–6.261). The basic activity of daily living in which a higher percentage of women were dependent was bathing, while for men it was going up and down stairs. Dependence on others for bathing was also the activity with the highest percentage of people when this analysis was performed in relation to educational level or income. The results according to sex, socioeconomic status, and educational level are shown for each activity of the Barthel index in Appendix 1 in Table A1.

### Barthel index activities and living arrangements in the community

Regarding the housing situation in the community, the activity most strongly associated with the loss of independence that comes with not being able to live independently and having to live with children, or other people besides one’s partner, was not being able to feeding independently (Table 3). Losing the ability to use the toilet independently was the second reason that made it necessary to live with other people. These activities were followed in order of frequency by being dependent on chair-to-bed transfers, having fecal incontinence, depending on others getting to dressing-undressing, and not being able to groom independently. Not being able to perform the rest of the activities on the Barthel index, or needing significant help from others to perform them, was not associated in this study with the need to live with other people.

**Table 3:** Analysis of the relationship between the level of dependence in performing each of the activities in the Barthel index and personal and social independence in the community, defined by housing situation (lives independently versus lives with children or other people), community living arrangements (lives confined versus can leave home to go for walks or engage in leisure activities), and the ability to leave home to go for walks or participate in leisure activities.

Barthel index: personal and social independence										
Barthel index activity	Activity	Housing situation		Statistical test	Leaving home		Statistical test	Leaves home for walks or leisure activities		Statistical test
	Level	Lives independently	Lives with other people	Odds ratio (Confidence interval)	Homebound	He leaves his house	Odds ratio (Confidence interval)	Yes	No	Odds ratio (Confidence interval)
Chair-to-bed transfers	Dependent	18.9% (10)	81.1% (43)	**4.300 (1.883–9.815)**	52.8% (28)	47.2% (25)	**0.266 (0.124–0.572)**	40.8% (20)	59.2% (29)	**0.265 (0.123–0.572)**
	Independent	50% (37)	50% (37)		23% (17)	77% (57)		72.2% (52)	27.8% (20)	
Up-down stairs	Dependent	34.2% (27)	65.8% (52)	1.375 (0.657–2.879)	45.6% (36)	54.4% (43)	**0.276 (0.118–0.644)**	50% (37)	50% (37)	**0.343 (0.154–0.762)**
	Independent	41.7% (20)	58.3% (28)		18.8% (9)	81.3% (39)		74.5% (35)	25.5% (12)	
Mobility on level surfaces	Dependent	21.3% (10)	78.7% (37)	0.354 (0.124–1.007)	38.2% (42)	61.8% (68)	0.346 (0.094–1.279)	39.1% (9)	60.9% (14)	**0.357 (0.140–0.909)**
	Independent	8.8% (7)	91.3% (73)		17.6% (3)	82.4% (14)		64.3% (63)	35.7% (35)	
Toilet use	Dependent	13% (3)	87% (20)	**4.888 (1.367–17.484)**	47.8% (11)	52.2% (12)	0.530 (0.212–1.323)	47.6% (10)	52.4% (11)	0.557 (0.216–1.436)
	Independent	42.3% (44)	57.7% (60)		32.7% (34)	67.3% (70)		62% (62)	38% (38)	
Dressing-undressing	Dependent	20% (8)	80% (32)	**3.250 (1.344–7.854)**	52.5% (21)	47.5% (19)	0.471 (0.218–1.019)	47.2% (17)	52.8% (19)	0.488 (0.221–1.077)
	Independent	44.8% (39)	55.2% (48)		29.9% (26)	70.1% (61)		64.7% (55)	35.3% (30)	
Grooming	Dependent	34% (16)	56.3% (45)	**2.491 (1.179–5.261)**	42.6% (26)	57.4% (35)	0.544 (0.261–1.136)	50% (28)	50% (28)	**0.477 (0.228–0.998)**
	Independent	66% (31)	43.8% (35)		28.8% (19)	71.2% (47)		67.7% (44)	32.3% (21)	
Bathing	Dependent	34.8% (32)	65.2% (60)	1.406 (0.634–3.114)	37% (34)	63% (58)	0.782 (0.341–1.793)	50% (17)	50% (17)	0.582 (0.261–1.296)
	Independent	42.9% (15)	57.1% (20)		31.4% (11)	68.6% (24)		63.2% (55)	36.8% (32)	
Feeding	Dependent	10.5% (4)	89.5% (34)	**7.945 (2.602–24.263)**	39.5% (15)	60.5% (23)	0.780 (0.356–1.709)	58.1% (50)	41.9% (36)	0.821 (0.366–1.842)
	Independent	48.3% (43)	51.7% (46)		33.7% (30)	66.3% (59)		62.9% (22)	37.1% (13)	
Bladder	Dependent	29.5% (13)	70.5% (31)	1.654 (0.757–3.615)	47.7% (21)	52.3% (23)	**0.446 (0.209–0.951)**	46.3% (19)	53.7% (22)	**0.440 (0.204–0.949)**
	Independent	41% (34)	59% (49)		28.9% (24)	71.1% (59)		66.3% (53)	33.8% (27)	
Bowels	Dependent	15.4% (4)	84.6% (22)	**4.077 (1.309–12.699)**	46.2% (12)	53.8% (14)	0.566 (0.236–1.360)	54.2% (13)	45.8% (11)	0.761 (0.309–1.873)
	Independent	42.6% (43)	57.4% (58)		32.7% (33)	67.3% (68)		60.8% (59)	39.2% (38)	

Cell color format: Mustard color: group 1 activities of the Barthel index. Green color: group 2 activities of the Barthel index. Blue color: group 3 activities of the Barthel index. Results that are statistically significant are shown in bold.

When these results were analyzed according to the percentage of people with severe dependency living with others, being dependent on others for feeding and toilet use remained the activities where the highest percentage of people living with others was observed. However, in order of frequency, the third activity in the Barthel index where dependence resulted in a higher percentage of people having to live with others was fecal incontinence, with dependence for chair-to-bed transfers in fourth place, followed by dependence on others for dressing-undressing.

Among people living independently in the community, the activities where the highest level of dependence was observed were bathing (34.8%) and going up and down stairs (34.2%), with the rest of the activities showing a lower level of dependence. In general, in this group living independently, at least seven out of ten people were independent when functional capacity was analyzed within each of the Barthel index activities.

However, among dependent people living with children or other people who were not their partners, individualized analysis of each activity in the Barthel index showed that at least one in two people in this situation was dependent on others to perform the activity analyzed. What is more, in the case of some activities, such as feeding (89.5%), toilet use (87%), chair-to-bed transfers (81.1%), and dressing-undressing (80%), it was observed that most people living with others were dependent on others to perform these activities.

Regarding bladder and bowel control, bowel incontinence (84.6%) and/or bladder incontinence (70.5%) were commonly present among people who had to live with their children or other people. This result contrasted with that observed among people who, despite having functional dependence, lived independently in the community, where these percentages were 15.4 and 29.5%, respectively.

On the other hand, when analyzing the manner of living in the community, not being able to leave the house to go for a walk or perform activities and being forced to live homebound was associated with not being able to independently perform chair-to-bed transfers and not being able to go up-down stair (Table 3). To a lesser extent, it was also associated with not being able to dress or undress and with urinary incontinence. Not being able to perform the rest of the Barthel index activities independently or with minimal assistance was not associated in this cohort with living homebound.

When analyzing these results using as a reference the percentage of people who were dependent on performing these activities, being dependent on performing the chair-to-bed transfers continued to be the situation that contributed to the highest percentage of people living homebound. Being dependent on dressing-undressing ranked second, urinary incontinence ranked third, and, among the activities that were significantly associated with community living, dependence on going up and down stair ranked fourth.

It was also observed that, among people living homebound (*n* = 45), 93.3% were dependent or needed help to walk. No other activity in the Barthel index had such a high percentage, as it was common for one in two people living homebound to be dependent on performing one of these activities. The activities in which people living homebound showed the least dependence were feeding and bathing (Table 3).

When both factors were analyzed together, living with children and living homebound, only dependence on one activity, chair-to-bed transfers, was associated with both situations.

Finally, functional dependence was likely to involve changes in how individuals interacted with their environment. Along these lines, we analyzed how functional dependence affected the ability to leave home independently, without the need for an escort, and to what extent it influenced having to do so accompanied by another person. The results of this analysis showed that when a person was dependent on others to perform any activity included in the Barthel index and lived homebound, the probability of leaving the house to go for a walk independently, without needing an accompanying person, was very low or nonexistent (Appendix 1 in Table A3). When this activity was performed, the presence of an accompanying person was the norm. However, when comparing performing the activity of leaving home accompanied versus being unable to perform it due to being homebound, dependence on a specific activity on the Barthel index did not always influence the outcome. In Group 1 activities, the level of dependence on chair-to-bed transfers (*χ*^2^ = 7.101; *p* = 0.007) and going up and down stairs (*χ*^2^ = 4.734; *p* = 0.029) was associated with being able to leave the house accompanied by another person, but dependence on others for walking or toilet use was not. In none of the Group 2 activities was the level of dependence for performing the activity associated with the ability to leave the house accompanied by another person. It was also observed that, among individuals who were independent in performing the activities of the Barthel index, leaving the house was an activity that was significantly more likely to be performed in the company of others (Appendix 1 in Table A3).

On the other hand, in addition to leaving home out of necessity, the extent to which these individuals left their homes without being obligated to do so, either for a walk or for leisure activities, was also analyzed. As a result of this analysis, it was observed that being dependent on any of the activities associated with mobility (chair-to-bed transfers, going up and down stairs, and walking) meant a lower probability of leaving home to go for a walk or enjoy leisure activities (Table 3). These two instrumental activities were also limited when the person was dependent on grooming or had urinary incontinence. Dependence on the other activities of the Barthel index was not associated in this study with a lower probability of leaving home to perform these activities.

Regarding the ability to independently purchase the products necessary for subsistence, without needing to be supervised or accompanied by another person, this activity was limited when there was dependence on others for transfers from chair to bed (OR 0.137; CI 0.030–0.624) and for going up and down stairs (OR 0.124; CI 0.038–0.402). It was also limited when the ability to bathe independently was limited (OR 0.166; CI 0.058–0.469), or when urinary incontinence was present (OR 0.188; CI 0.041–0.858). The remaining results for this activity are presented in Appendix 1 in Table A4.

Finally, 100% of people who were dependent on others to use the toilet, or who had fecal incontinence, did not retain the ability to make the necessary purchases for their subsistence under the supervision of another person. It was also observed that only one in ten still retained the ability to make these purchases independently. On the other hand, being dependent on others for bathing or having urinary incontinence was also associated with a lower probability of being able to make these purchases under the supervision of another person (Appendix 1 in Table A4).

The Barthel index activity, whose dependence most limited the performance of instrumental activities associated with maintaining personal and social independence, was dependent on chair-bed transfers. This was followed by dependence on going up and down stairs, dressing and undressing, and toilet use.

### Barthel index activities and use of assistive mobility devices

In people with functional dependence, mobility can be achieved with help from assistive devices. The use of these devices was analyzed in relation to the dependence these people had on performing the different activities included in the Barthel index (Appendix 1 in Table A5).

In relation to wheelchair use (Table 4), the Barthel index activity most strongly associated with the need to use a wheelchair was going up-down stair. In descending order of frequency, this was followed by dependence on transfers between chair and bed, inability to dress or undress, inability to grooming independently, inability to use the toilet independently, and fecal incontinence. Dependence on the other activities in the Barthel index was not associated with the need to use a wheelchair.

**Table 4:** Analysis of the relationship between the level of dependence for performing each activity included in the Barthel index, the use of a wheelchair as a mobility aid, and the availability of caregivers hired by dependent individuals or their families to perform household tasks and personal care at the dependent person’s home.

Barthel index activities, wheelchair use and availability of caregivers										
Barthel index activity	Activity	Wheelchair		Statistical test	Private caregiver		Statistical test	Internal caregiver		Statistical test
	Level	Yes	No	Odds ratio (Confidence interval)	Yes	No	Odds ratio (Confidence interval)	Yes	No	Odds ratio (Confidence interval)
Chair-to-bed transfers	Dependent	49.1% (26)	50.9% (27)	**3.788 (1.733–8.278)**	39.6% (21)	60.4% (32)	1.285 (0.614–2.688)	22.6% (12)	77.4% (41)	1.463 (0.599–3.575)
	Independent	20.3% (15)	79.7% (59)		33.8% (24)	66.2% (47)		16.7% (12)	83.3% (60)	
Up-down stairs	Dependent	43% (34)	57% (45)	**4.425 (1.769–11.071)**	41.8% (33)	58.2% (46)	1.973 (0.888–4.381)	17.7% (14)	82.3% (65)	0.775 (0.313–1.922)
	Independent	14.6% (7)	85.4% (41)		26.7% (12)	73.3% (33)		21.7% (10)	78.3% (36)	
Mobility on level surfaces	Dependent	34.5% (38)	65.5% (72)	2.882 (0.782–10.628)	39.8% (43)	60.2% (65)	**0.216 (0.047–0.998)**	20.9% (23)	79.1% (87)	0.270 (0.034–2.163)
	Independent	17.6% (3)	82.4% (14)		12.5% (2)	87.5% (14)		6.7% (1)	93.3% (14)	
Toilet use	Dependent	52.2% (12)	47.8% (11)	**2.821 (1.120–7.105)**	52.2% (12)	47.8% (11)	2.248 (0.898–5.628)	39.1% (9)	60.9% (14)	**3.729 (1.371–10.143)**
	Independent	27.9% (29)	72.1% (75)		32.7% (33)	67.3% (68)		14.7% (15)	85.3% (87)	
Dressing-undressing	Dependent	52.5% (21)	47.5% (19)	**3.703 (1.669–8.212)**	50% (20)	50% (20)	**2.360 (1.086–5.130)**	30% (12)	70% (28)	**2.607 (1.048–6.484)**
	Independent	23% (20)	77% (67)		29.8% (25)	70.2% (59)		14.1% (12)	85.9% (73)	
Grooming	Dependent	44.3% (27)	55.7% (34)	**2.950 (1.356–6.414)**	41.7% (25)	58.3% (35)	1.571 (0.752–3.283)	29.5% (18)	70.5% (43)	**4.047 (1.482–11.050)**
	Independent	21.2% (14)	78.8% (52)		31.3% (20)	68.8% (44)		9.4% (6)	90.6% (58)	
Bathing	Dependent	35.9% (33)	64.1% (59)	1.888 (0.770–4.627)	44.9% (40)	55.1% (49)	**4.898 (1.740–13.785)**	24.2% (22)	75.8% (69)	**5.101 (1.130–23.024)**
	Independent	22.9% (8)	77.1% (27)		14.3% (5)	85.7% (30)		5.9% (2)	94.1% (32)	
Feeding	Dependent	39.5% (15)	60.5% (23)	1.580 (0.714–3.499)	57.9% (22)	42.1% (16)	**3.766 (1.689–8.396)**	34.2% (13)	65.8% (25)	**3.593 (1.430–9.028)**
	Independent	29.2% (26)	70.8% (63)		26.7% (23)	73.3% (63)		12.6% (11)	87.4% (76)	
Bladder	Dependent	43.2% (19)	56.8% (25)	2.107 (0.975–4.553)	50% (22)	50% (22)	**2.478 (1.154–5.320)**	27.3% (12)	72.7% (32)	2.156 (0.874–5.321)
	Independent	26.5% (22)	73.5% (61)		28.8% (23)	71.3% (57)		14.8% (12)	85.2% (69)	
Bowels	Dependent	50% (13)	50% (13)	**2.607 (1.077–6.308)**	53.8% (14)	46.2% (12)	**2.522 (1.045–6.084)**	26.9% (7)	73.1% (19)	1.777 (0.646–4.888)
	Independent	27.7% (28)	72.3% (73)		31.6% (31)	68.4% (67)		17.2% (17)	82.9% (82)	

Cell color format: Mustard color: group 1 activities of the Barthel index. Green color: group 2 activities of the Barthel index. Blue color: group 3 activities of the Barthel index. Results that are statistically significant are shown in bold.

According to the Barthel index activity that determined its use, half of the people who were dependent for chair-to-bed transfers used a wheelchair, and this situation resulted in 63.4% of wheelchair users also being dependent for these transfers.

Similarly, among people who were dependent on others for going up and down stairs, 43% were wheelchair users. However, among wheelchair users, 82.9% were dependent on other people for going up-down stairs.

Regarding dressing-undressing, half of the people who were dependent in this activity were also wheelchair users, and this percentage was also the same for wheelchair users who were dependent in dressing-undressing.

This situation was like the one observed among people who needed other people to help with grooming, where 44.3% were wheelchair users. In turn, two out of three wheelchair users were dependent on other people for grooming.

However, this contrasted with the situation of people who were dependent on others to use the toilet. Although half of this group used wheelchairs, only 29.3% of wheelchair users reported being dependent on others to use the toilet.

Finally, half of the people with fecal incontinence were also wheelchair users, but among wheelchair users, only 31.7% had fecal incontinence.

In relation to the use of a walker (Appendix 1 in Table A5), this mobility aid was not associated with dependence on any of the Barthel index activities. Regarding the use of a walker, this mobility aid was not associated with dependence on any of the Barthel index activities. The Barthel index activity in which dependence on a walker was most strongly associated with an increased need to use one was going up and down stair. This was followed, in order of frequency, by being dependent on assistance for chair-to-bed transfers and for walking independently. That is, the activities included in group 1, in which the mobility factor was clearly relevant.

Analyzing these results globally, in activities where differences in the use of mobility aids were significant, the use of a wheelchair was associated with a higher level of dependence on that activity, while when the person was independent for that activity, the prevailing outcome was not the use of any mobility aids, but rather the use of crutches-cane. People dependent on toilet use were the least likely to use crutches-cane, with eight out of ten people dependent on this activity not using this mobility aid. A similar result was observed among people dependent on chair-to-bed transfers or dressing-undressing. Also, most people who are dependent on others for feeding, grooming, or going up and down stairs, or who had urinary incontinence, did not use crutches-cane (Appendix 1 in Table A5).

### Barthel index activities and availability of assistants for domestic tasks and personal care, or an internal caregiver

Functional dependence often involves the need for assistance from others in performing basic activities of daily living.

Having a public assistant to help perform domestic tasks or personal care was not associated with dependence on any of the activities included in the Barthel index. When analyzing the availability of this type of assistant among people who were dependent on others to perform the activity, the activity where their presence was most frequent was using the toilet. In contrast, the activity where dependence was least frequently assisted by a public assistant was feeding (Appendix 1 in Table A6).

Regarding other types of assistants or caregivers (Table 4), not having control over the sphincters was associated with a higher probability of having a private caregiver. The presence of this type of private caregiver was significantly associated with the level of dependence on bathing, feeding, and dressing-undressing. Finally, the presence of an internal caregiver was associated, in order of frequency, with dependence on bathing, toilet use, feeding, and dressing-undressing (Table 4).

At the same time, and in contrast to the results observed with public assistants, the activity that required a higher percentage of private caregivers was feeding. Also, paradoxically with respect to what was expected, the activities where it was less frequent to have a private caregiver when one was dependent on performing that activity were chair-to-bed transfers and walking, both associated with mobility (Table 4). None of the activities associated with mobility (chair-to-bed transfers, going up and down stairs, walking) were associated with a higher level of dependency on a public assistant or internal caregiver. Only dependency on walking was associated with having a private caregiver.

On the other hand, the activity in which dependence was most strongly associated with having an internal caregiver was dependence on toilet use. However, the three activities in which mobility was essential for their performance showed that dependence on their performance was associated with lower percentages of availability of this support resource represented by the internal caregiver, compared to those observed for the rest of the activities (Table 4).

Finally, the results showed that almost half of the people who were dependent on each of the Barthel index activities analyzed did not have a social services-assigned assistant for domestic tasks. However, it was also observed that, except for dependence on walking, going up-down stairs, and bathing, for the rest of the activities it was more common to have a public assistant for domestic tasks when the person was independent or needed minimal help to perform that activity (Appendix 1 in Table A6).

### Interdependence between Barthel index activities

The presence of dependence for other activities in the Barthel index when dependent for a specific activity was also analyzed (Table 5). The Barthel index activities that presented the highest percentages of codependence were bathing, chair-to-bed transfers, and going up and down stairs. The lowest percentages of codependence were observed for fecal incontinence, with codependence when dependent on toilet use coming in second place. However, when this parameter was assessed within the context of dependence on another activity, it was observed that this codependence on toilet use frequently reached a percentage of 100%.

**Table 5:** Interdependence between Barthel index activities. Analysis between dependent individuals for each activity.

Interdependence between Barthel index activities										
Activity	Chair-to-bed-transfers	Up-down stairs	Mobility on level surfaces	Toilet use	Dressing-undressing	Grooming	Bathing	Feeding	Bladder	Bowels
Chair-to-bed transfers	–	58.2% (46)	80.8% (21)	91.3% (21)	90% (36)	68.9% (42)	51.1% (47)	76.3% (29)	75% (33)	76.9% (20)
Up-down stairs	86.8% (46)	–	96.2% (25)	100% (23)	90% (36)	82% (50)	69.6% (64)	81.6% (31)	93.2% (41)	88.5% (23)
Mobility on level surfaces	39.6% (21)	31.6% (25)	–	47.8% (11)	42.5% (17)	29.5% (18)	26.1% (24)	44.7% (17)	40.9% (18)	34.6% (9)
Toilet use	39.6% (21)	29.1% (23)	42.3% (11)	–	55% (22)	36.1% (22)	100% (23)	42.1% (16)	52.3% (23)	73.1% (19)
Dressing-undressing	67.9% (36)	45.6% (36)	65.4% (17)	95.7% (22)	–	60.7% (37)	97.5% (39)	73.7% (28)	68.2% (30)	73.1% (19)
Grooming	79.2% (42)	63.3% (50)	69.2% (18)	95.7% (22)	92.5% (37)	–	57.6% (53)	81.6% (31)	75% (33)	76.9% (20)
Bathing	88.7% (47)	81% (64)	92.3% (24)	100% (23)	97.5% (39)	86.9% (53)	–	92.1% (35)	93.2% (41)	92.3% (24)
Feeding	54.7% (29)	39.2% (31)	65.4% (17)	69.6% (16)	70% (28)	50.8% (31)	38% (35)	–	54.5% (24)	53.8% (14)
Bladder	62.3% (33)	51.9% (41)	69.2% (18)	100% (23)	75% (30)	54.1% (33)	44.6% (41)	63.2% (24)	–	88.5% (23)
Bowels	37.7% (20)	29.1% (23)	34.6% (9)	82.6% (19)	47.5% (19)	32.8% (20)	26.1% (24)	36.8% (14)	52.3% (23)	–

Rows: Percentage of people who are dependent on the activity listed in the column out of the total number of people dependent on the activity listed in the row. Columns: Percentage of people who are dependent on the activity listed in the row out of the total number of people dependent on the activity listed in the column. Cell color format: Mustard color: group 1 activities of the Barthel index. Green color: group 2 activities of the Barthel index. Blue color: group 3 activities of the Barthel index.

Analyzing the results in relation to the groups created in the design of this study, in relation to the participation of mobility in the performance of each activity, 8.7% (*n* = 11) of the people in this cohort were dependent on the four activities included in group 1, 28.3% (*n* = 33) were dependent on the three activities in group 2, and 11% (*n* = 14) were dependent for the three activities in group 3. At the same time, 31.5% (*n* = 40) were independent for the four activities in group 1, 21.3% (*n* = 27) for the three activities in group 2, and 52% (*n* = 66) for those in group 3.

When this analysis was performed considering dependence on at least one activity included in each group, but not in all of them, 72.4% (*n* = 92) of the individuals in this cohort were dependent in at least one activity in group 1, and 91.3% (*n* = 116) were independent in at least one activity in this group. Regarding group 2, 78.7% (*n* = 100) were dependent on at least one activity, and 71.6% (*n* = 91) were independent in at least one activity. Finally, in group 3, 48% (*n* = 61) were dependent on at least one activity and 89% (*n* = 113) were independent in at least one activity.

Dependence on others for feeding, which involved the need to hire a private caregiver or internal caregiver, was not one of the activities most frequently listed as dependence associated with dependence on another activity. However, when people were dependent on eating, a high percentage of them were also co-dependent on other activities. However, the activity of washing, which was also involved in this need for hired caregivers, showed an important level of co-dependence in both situations.

### Ability to perform each activity on the Barthel index and five-year survival

The majority of Barthel index activities were consistently associated with the probability of survival at 5 years, such that being dependent on performing them implied a lower probability of survival (Appendix 1 in Table A7; Appendix 2 in Tables S1–S3). Only urinary incontinence was not associated with this lower probability of survival. In this cohort from the Orcasitas neighborhood of Madrid (Spain), the activities in which dependence was most highly associated with a lower probability of survival were the ability to use the toilet, the ability to dress and undress, the ability to walk independently, the ability to perform chair-to-bed transfers, and the ability to go up and down stairs.

At the same time, the results were analyzed in relation to the groups of activities established in this study based on the criterion of mobility, using two criteria: 1: Being dependent for all activities included in the group versus being independent for all of them. 2: Being dependent on at least one of the activities in the group versus being independent for at least one activity in that group (Appendix 1 in Table A8). Using the first criterion, the probability of survival in this cohort was lower for people who were dependent for all the Barthel index activities included in group 1, which included Barthel index activities where postural balance played a role, but mobility was the essential requirement for their performance. Second place was occupied by the activities included in group 2, which included Barthel index activities where mobility and postural balance contributed to their performance in an equivalent way. In this group 2 mobility was necessary, but its limitation did not totally condition their execution. Last place was occupied by group 3, which included Barthel index activities where mobility was not an essential criterion for their completion, a group in which urinary incontinence, in isolation, was not associated with a lower probability of survival.

Using the first criterion, within group 1 it was observed that all the people who were dependent for the four activities in group 1 died during the 5 years of follow-up, while among those who were independent for this set of activities, 42.5% died. When survival in group 2 was analyzed, being dependent for their three activities was associated with a mortality of 91.7%, while those who were independent for all activities in group 2 had a mortality of 40.7%. Finally, within group 3, 85.7% of those who were dependent for their three activities died, while 48.5% of those who were independent for these three activities died.

Simultaneously, using the second criterion, within group 1, 70.1% of those who were dependent in at least one activity died, as did 57.8% (*n* = 67) of those who were independent in at least one activity. In group 2, these results were 67% (*n* = 67) among those who were dependent in at least one activity and 49.5% (*n* = 45) among those who were independent in at least one activity in this group 2. Finally, in group 3, during the five-year follow-up period, 75.4% of those who were dependent in at least one activity in this group died, and 58.4% among those who were independent in at least one activity in this group 3. Using this second criterion reversed the order of mortality observed when using the first endpoint.

In the survival analysis using Cox regression (Table 6), it was observed that the activity in which dependence on others for its performance implied a higher risk of mortality was dependent on dressing-undressing (HR 2.842; CI 1.806–4.471). This risk was increased in all Barthel index activities, except for urinary incontinence, duplicating the probability of death during this follow-up period when the patient was dependent for activities such as toilet use, going up-down stair, bathing, and chair-to-bed transfers.

**Table 6:** Survival analysis using Cox regression.

Cox regression, 5-year survival								
Variable	B	ET	Wald	gl	Sig.	Exp(B)	CI 95, 0% for Exp(B)	
							Lower	Upper
Chair-to-bed transfers	0.844	0.229	13.519	1	0.001	2.325	1.483	3.645
Up-down stair	0.891	0.260	11.727	1	0.001	2.438	1.464	4.061
Mobility on level surfaces	0.566	0.261	4.,720	1	0.030	1.761	1.057	2.935
Toilet use	0.909	0.260	12.278	1	0.001	2.483	1.493	4.129
Dressing-undressing	1.044	0.231	20.390	1	0.001	2.842	1.806	4.471
Bathing	0.876	0.296	8.767	1	0.003	2.401	1.345	4.288
Grooming	−0.620	0.230	7.285	1	0.007	0.538	0.343	0.844
Feeding	0.692	0.235	8.688	1	0.003	1.997	1.261	3.163
Bladder	−0.262	0.160	2.664	1	0.103	0.770	0.562	1.054
Bowels	0.564	0.257	4.832	1	0.028	1.758	1.063	2.907

Hazard ratios and confidence intervals for each Barthel index activity. Cell color format: Mustard color: group 1 activities of the Barthel index. Green color: group 2 activities of the Barthel index. Blue color: group 3 activities of the Barthel index.

By linear regression, the percentage of survival variance explained by dependence on each Barthel index activity was estimated (Appendix 1 in Table A9). It was observed that dependence on dressing and undressing explained the highest percentage of this variance (12.5%) in this cohort. No other Barthel index activity exceeded 10%. The Barthel index activities explained 14.8% of the variance in survival.

Finally, the discriminatory power of each Barthel index activity in relation to survival was analyzed using ROC curves. This analysis was performed by grouping the activities into the three operational classification groups described above.

**Figure 1:**
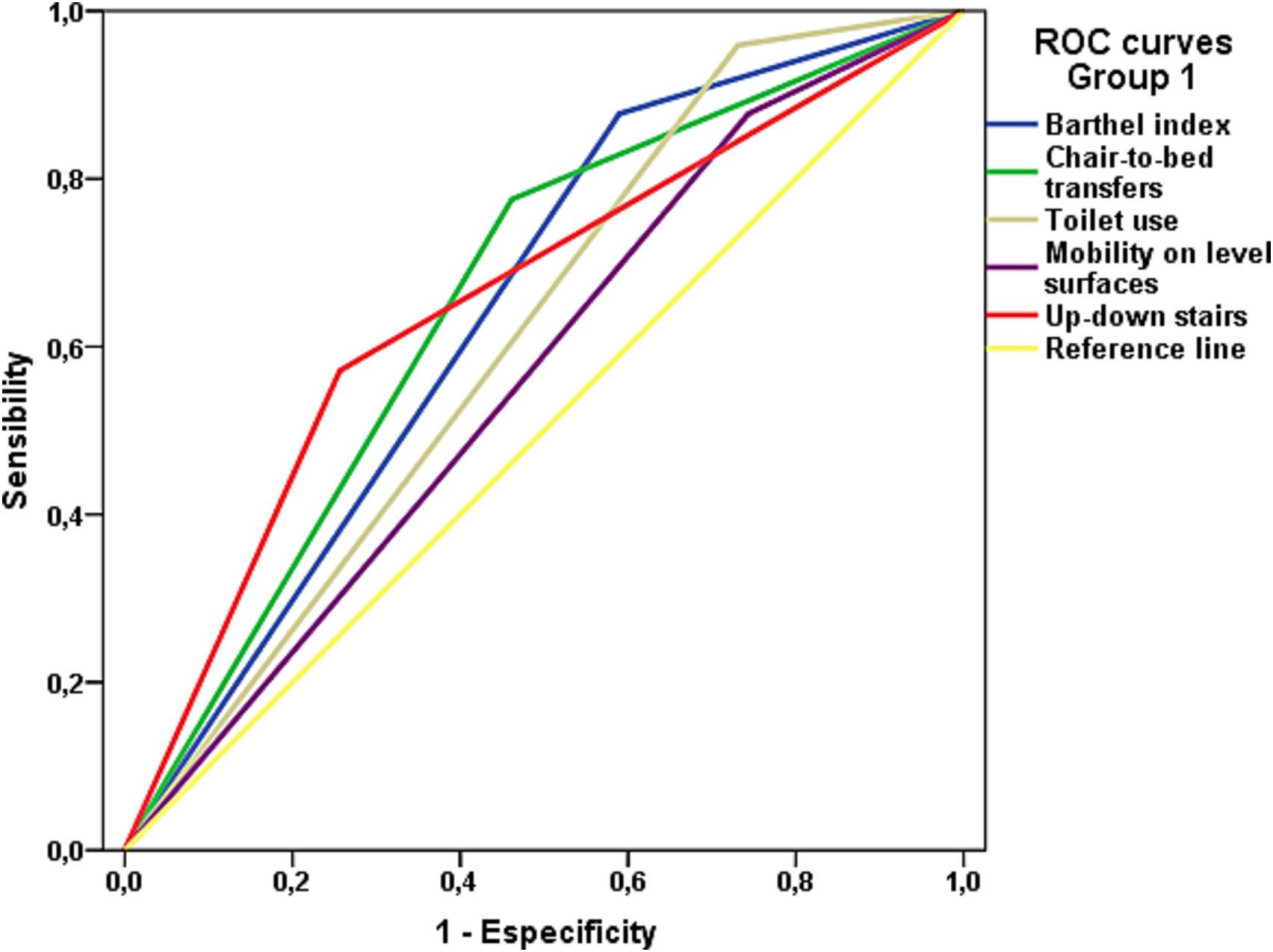
Analysis of the capacity for discrimination against Barthel index activities in which mobility is a necessary factor for their performance as a test to assess survival (group 1). Comparison with the Barthel index.

Regarding group 1, activities in which mobility played a relevant role in their performance, it was observed (Figure 1) that, with respect to the established gold standard (the area under the ROC curve of the Barthel index), the activities of chair-bed transfers and going up and down stairs had a slightly higher discriminatory capacity than the gold standard. However, this was not observed regarding the ability to walk independently. Using the DeLong method, the differences were significant, showing that both the Barthel index and the activities of chair-to-bed transfers, going up-down stair, and toilet use discriminated against better probability of survival than the activity of walking (Appendix 3 in Figure S1).

**Figure 2:**
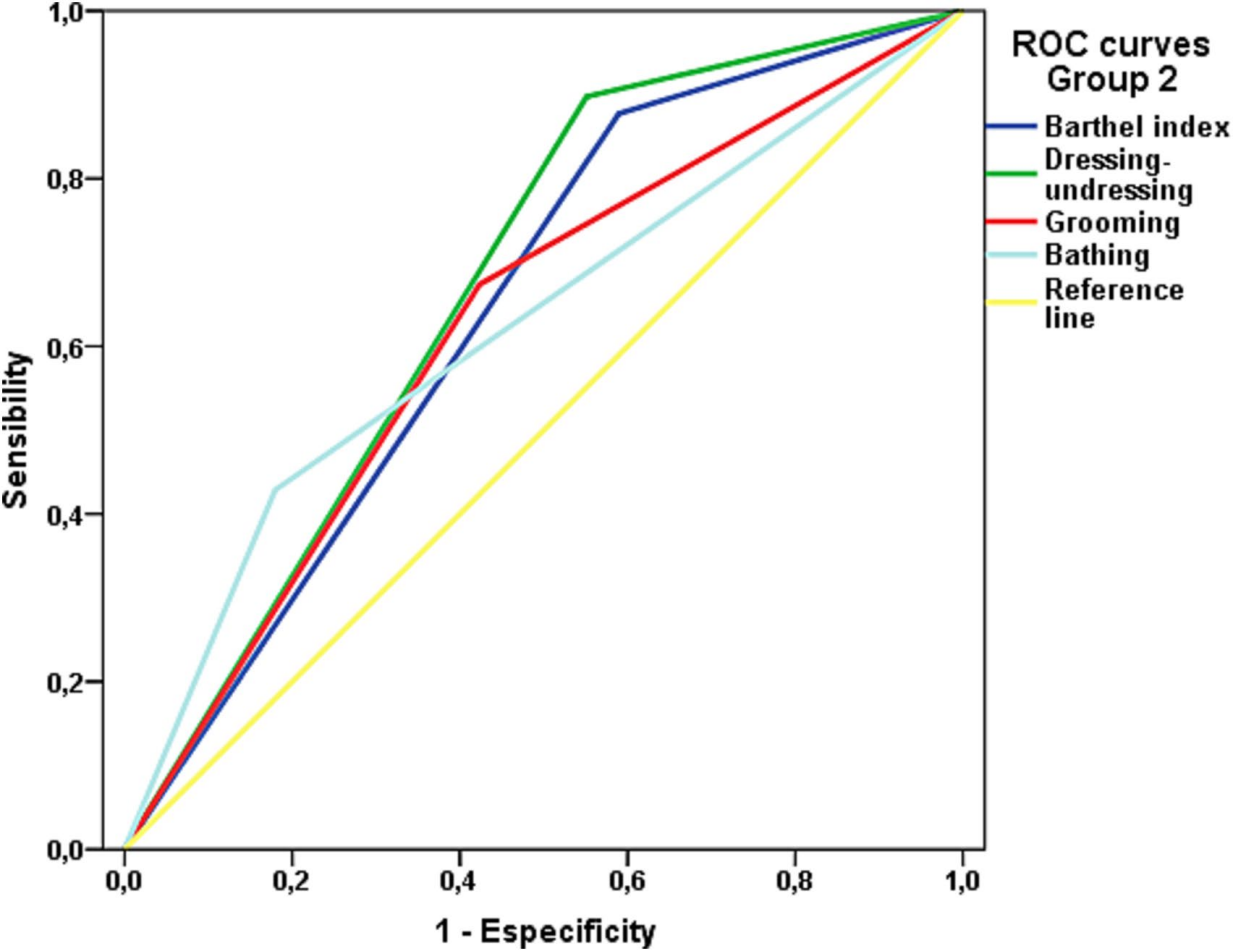
Analysis of the discriminatory capacity of Barthel index activities in which mobility is associated but not essential factors for their performance, as a test to assess survival (group 2). Comparison with the Barthel index.

The results in group 2, Barthel index activities where mobility was an associated factor for their performance but not essential (Figure 2), showed a discriminatory capacity as a test like that of the Barthel index, slightly better when considering the activity of dressing-undressing. However, in any case, and as with the Barthel index itself, this ability to discriminate survival probability could be classified as poor. The differences between these activities and the Barthel index using the DeLong method were not significant, indicating that the ability to discriminate as a survival test was similar between them (Appendix 3 in Figure S2).

**Figure 3:**
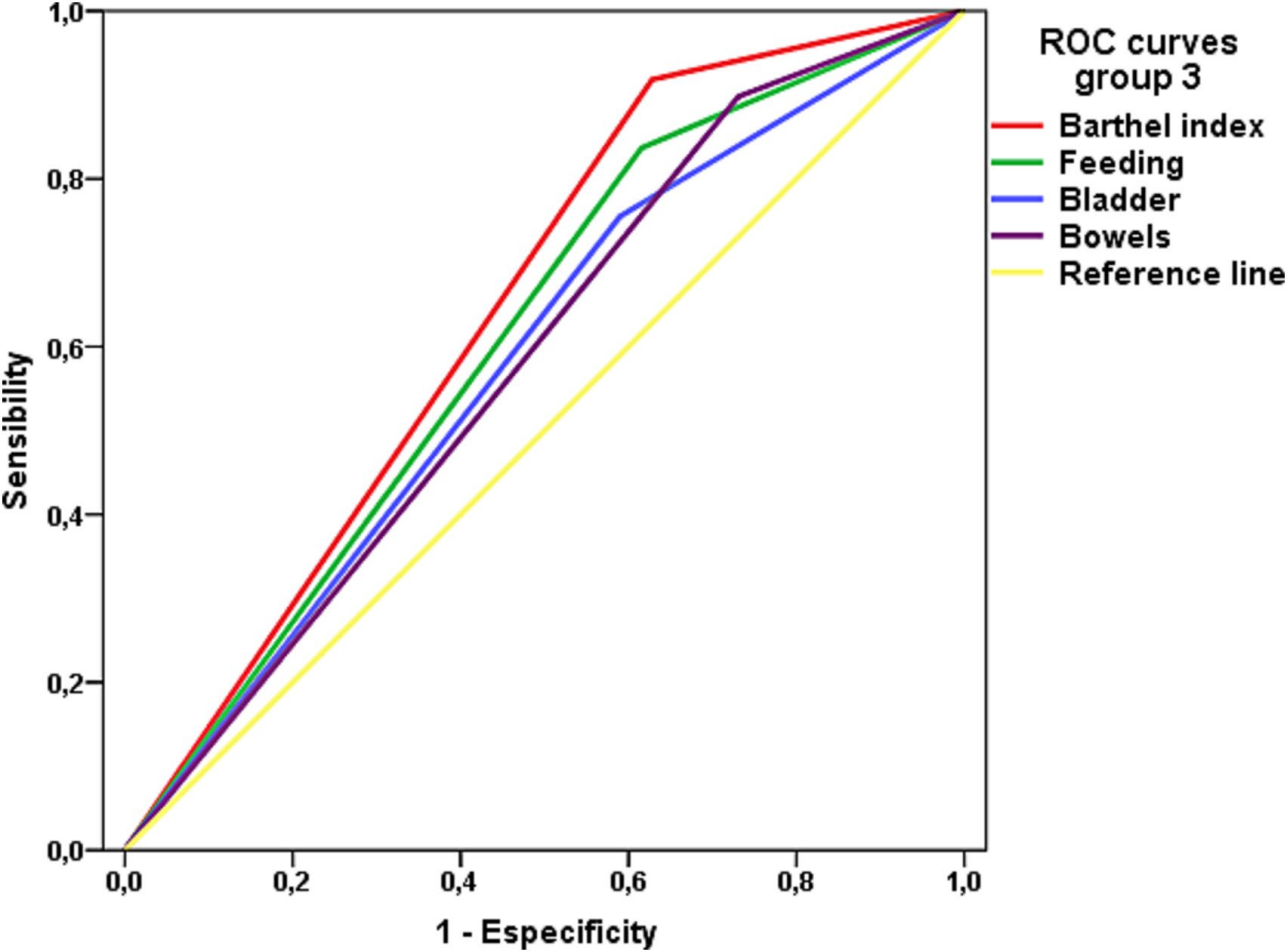
Analysis of the discriminatory capacity of Barthel index activities that do not require mobility as a necessary factor for their performance as a test to assess survival (group 3). Comparison with the Barthel index.

Finally, with regard to the Barthel index activities included in group 3, which were those where mobility was not necessary for their performance or played a minimal role (Figure 3), the ROC curve analysis showed that the discriminatory power as a test for estimating survival of the bladder and anal continence control factor was low, and these tests were not suitable for this analysis in this population. Using the DeLong method, no significant differences were observed between the activities compared in terms of this discrimination capacity (Appendix 3 in Figure S3).

## Discussion

Within a hypothesis-generating observational framework, this study yielded four main findings. The first is that existing geriatric theories may contain gaps that affect the characterization of the initial stages of functional dependence. Second, functional dependence occurs within a context in which functional status and environment establish positive and negative feedback loops, ultimately generating a dynamic network that, although specific to each individual, tends to form patterns among people with similar characteristics and has a social component that cannot be ignored. The third finding is that mortality in this cohort increased as the number of activities on the Barthel Index for which individuals were dependent increased, with dependence on one activity, dressing and undressing, and a specific co-dependence, toilet use and bathing, being associated with the highest mortality risks. And finally, the fourth finding proposes the hypothesis that the assessment of the mobility domain of intrinsic ability would likely benefit from expanding its evaluation through other indicators that allow for a greater degree of differentiation once functional dependence has already been established.

Although functional dependence seems to function as a global entity that leads to poorer outcomes in health, quality of life, and mortality, in truth it is made up of multiple facets that determine the final outcomes. These facets often respond to personal characteristics, on which social and environmental factors interact (29, 32, 33). In an attempt to delve deeper into the behavioral patterns and networks established by these facets, the results of this study show that the personal and social environment of dependent individuals is influenced differently depending on the activity and the level of dependency observed in the performance of each of the activities of daily living analyzed. These differences suggest associations and profiles that ultimately identify more complex patients within each group of activities analyzed. The absence of a uniform pattern regarding dependency highlights the complexity of the interrelationships that arise within the framework of functional dependency.

The analysis of these interrelationships has clinical relevance, as it allows for the formulation of hypotheses regarding which activities may have the greatest impact on a person’s overall dependency, comorbidity, and survival. In this regard, Farrokhi et al. (8) observed that, in hemodialysis patients, evaluating only four activities of daily living, rather than the ten included in the Barthel index, yielded similar results. Boström et al. (9) also observed that not all activities in the Barthel index were associated with the development of depression. On the other hand, Fong et al. (18) found that dependence in bathing was the strongest predictor of admission to a nursing home, followed by dependence in dressing-undressing and feeding. Finally, Gloystein et al. (7) suggest that the Barthel index score and the differences observed among the activities it includes may serve as a good basis for case management and healthcare planning.

These interrelationships also have a social component that must be considered. In this study, greater overall dependence implied a denser network and a greater need for care, which translated into a higher probability of living with one’s children, as also noted in the study by Oliveria et al. (25), and a greater need for caregivers at home, a situation already reflected in studies such as that by Díaz-Venegas et al. (29). Furthermore, as Redzovic et al. (32) also suggest in their study, the patterns established by these interrelationships in a population group of such advanced age and with low educational and socioeconomic status create a framework for proposing measures that promote healthy aging, with this study providing potential targets for action.

On the other hand, regarding the classical model, which establishes that loss of mobility is one of the pillars underpinning functional dependence (6–12), these interrelationships raise the hypothesis that other patterns or sequences of combinations might exist, as Khalili et al. (30) also note, or that even this initial pattern in which loss of mobility ultimately leads to functional dependence might have a preceding stage that has not yet been adequately characterized. In this regard, Gloystein et al. (7) noted that dependence in dressing-undressing and bathing were among the most common reasons for not achieving the maximum score on the Barthel index.

The duration of this preceding stage is currently difficult to establish, a point also noted by Khalili et al. (30). The incipient development of dependence on others to perform activities such as dressing-undressing, grooming, and bathing would represent an initial stage, which could go unnoticed, as it is often associated by professionals and family members with age-related declines in abilities, until the transition to other states occurs. Normalizing its presence and rendering invisible its potential impact on the development of functional dependence until limitations in mobility become evident (30). In this regard, Ray et al. (5) report that not all activities included in the Barthel index have received the same level of attention in research, noting that, specifically, the activities of grooming, dressing-undressing, and bathing are those that had the fewest studies in their review of the existing literature. This lack of research also contributes to potential gaps in understanding the impact of dependence on these activities on overall functional dependence and on the timeline of the development of this dependence, with the present study offering hypotheses regarding this timeline and impact.

This hypothesis would be supported by the observed result that one in four people in this cohort were dependent on assistance for all three activities included in Group 2 (dressing-undressing, grooming, bathing), while only one in ten people were dependent on assistance for all four activities in Group 1 (walking independently, chair-to-bed transfers, going up-down stairs, and toilet use) activities in which mobility was an essential requirement for their performance; and also only one in ten people were dependent for the three activities in Group 3 (feeding, or maintaining anal and bladder control), activities associated with self-care. The marked differences in prevalence between Group 2 and Groups 1 and 3 suggested a different timeline for the cohort to reach this overall level of dependence, a timeline that other studies, such as that by Sagari et al. (13), also demonstrated, or that the studies by Khalili et al. (30) and Levy et al. (31) presented with a similar pattern. The activities in group 2 required a certain degree of balance and mobility to perform, but they may also be activities where personal self-adaptations or small modifications to the environment can minimize their impact, helping to make the limitations in these functional areas less visible.

Our finding that dependence on Group 2 activities was more prevalent than in Group 1 is consistent with the hierarchical model proposed by Jagger et al. (33) and Sagari et al. (13) and is also in line with the results observed in the studies conducted by Levy et al. (31) and Redzovic et al. (32). This consistency with other studies would support the suggestion that, when referring to activity groups, dependence on Group 2 activities likely emerges earlier than dependence on activities included in the other two groups. Alternatively, it could also be argued that these activities are more likely to generate dependence in the early stages of functional decline, whereby, as suggested by Khalili et al. (30), establishing typical patterns of disability that could be useful in medical decision-making. The parallel observation that, in the opposite situation, only one in five people in this cohort was simultaneously independent for dressing-undressing, grooming, and bathing, the activities in Group 2, while for the activities in group 1 one in three people was independent and for those in Group 3 one in two, would also support this approach. Other studies, such as the one conducted by Abizanda et al. (11) have observed in the longitudinal follow-up of patients over 70 years of age that the loss of mobility is greater than the loss of ability to perform basic activities of daily living, and that, at the start of these authors’ study, the loss of ability to perform the activities we included in Group 2 was much more prevalent than those we included in Group 1, confirming the results observed in our study in a population from the same country.

Regarding the clinical implications of the findings observed in this study, and in relation to survival, although numerous studies have analyzed the sequences that occur during functional decline (13, 16, 30, 31, 33), few have addressed the effect of these sequences (34), or of dependence on others to perform each activity, on survival (11). The results of this study show that the potential pattern of deterioration and the differences between groups did not imply that mortality was significantly higher in people dependent for activities in Group 2, compared to the other groups, being high and similar in all three activity groups.

However, this group outcome had nuances when each activity was analyzed individually. Among the five activities in the Barthel index whose dependence was associated with higher mortality, four were activities in Group 1 (walking independently, chair-to-bed transfers, going up-down stairs, and toilet use) and one (dressing and undressing) was in Group 2. As noted in the study by Wang et al. (35), limited mobility is a predictor of mortality, and mobility is a fundamental component of intrinsic capacity. However, this would only partially explain the findings regarding the activity of dressing-undressing, which was the activity most strongly associated with mortality risk in this study.

Several mechanisms could explain why dependence on assistance with dressing-undressing was the strongest predictor of mortality. This activity requires mobility; however, dependence on assistance with this activity did not show very high rates of co-dependence with the ability to walk. However, it did show high rates of codependence with grooming and bathing, the other two activities in Group 2, activities in which dependence was also a predictor of mortality risk. This result reinforced the entity of this group as a distinct category and pointed toward a possible causal mechanism. Within a possible multifactorial origin, where sensory impairment (vision, hearing), loss of strength, and loss of muscle mass could be involved, balance likely played a role. Mobility alone did not explain this result, and, consistent with the findings of Heiland et al. (14) regarding activities such as dressing-undressing, bathing, and toilet use, balance played a fundamental role in the ability to perform these tasks. This indicates that these individuals’ intrinsic capacity is influenced by factors beyond the mere ability to walk and that, in functional assessment, these nuances have clinical relevance for establishing subgroups and planning specific activities.

In addition, other aspects needed to be addressed. In the analysis of associations between categorical variables, the probability of the “mortality” event occurring was higher among those who were dependent in the activity of toilet use than in the other activities. As was the case with dependence on dressing-undressing, this activity, which also requires mobility, did not show high percentages of codependence with the ability to walk. However, it did show such percentages with the activities of going up and down stairs and chair-to-bed transfers, the other two activities in Group 1, and, above all, with the activity of washing, from Group 2. This last codependency, bathing/toilet use, was the only one that reached 100% in both directions. Balance likely also played a role in this result, within the possible multifactorial origin posited as the main explanatory hypothesis.

Limitations in performing these activities of daily living resulting from balance impairments have been examined in several studies. Heiland et al. (14) observed that individuals with balance impairments had limited ability to perform activities such as dressing-undressing, bathing, and toilet use, and that this finding was independent of cognitive status and associated comorbidities. King et al. (36) also observed this relationship between balance and the ability to perform these three activities.

On the other hand, it was also observed that dependence in one activity, without necessarily being dependent in the rest of the activities in that group, was also associated with a lower probability of survival at 5 years. This result suggested that there was a gradient in mortality, from being independent in all activities in one of the groups created to being dependent in all activities in that group, with the risk of mortality increasing progressively as the number of activities in which the person was dependent increased.

This entire framework ultimately took the form of a network, which included, as also observed in the studies by Redzovic et al. (32) and Oliveira et al. (25), the social implications of the findings observed in this study. The decline in the ability to perform activities such as dressing-undressing, grooming, and bathing has been associated by Greiman et al. (37) with a parallel loss of social autonomy, referring to the ability to leave the house and/or engage in leisure activities. These activities are also associated with the maintenance of personal identity, the loss of which, once established, as noted by Khalili et al. (30), tends to persist over time. On the other hand, this independence-dependence gradient, which previously influenced survival, showed that people living independently had a better functional profile than those living with their children or with other people. Among those living independently, only one in three was dependent on bathing or going up-down stair, with lower percentages of dependency for other activities.

Furthermore, within the nodes that made up the network around dependency, the loss of personal and social independence associated with functional dependence could be reflected in multiple ways, one of the most significant being the loss of the ability to live independently. Having to live with other people depends of multiple factors (13, 24, 25, 29, 38, 39), and functional dependence is one of the most relevant among them. In this cohort, being unable to perform chair-to-bed transfers independently, depending on others to feed, grooming, dressing-undressing, and toilet use, or having bowel incontinence was associated with the housing situation of having to live with children. The association between greater functional impairment and the need to live with other people or be institutionalized had already been described (13, 24, 25, 29, 39–41), with these results providing information on the activities on which this association may be based. On the other hand, it should also be borne in mind that living with children does not always respond to the parents’ own functional limitations. Today, the economic difficulties of children, or the absence of a partner or their own home, force these children to return to their parents’ home, or never to leave it (25, 42).

At the same time, within this network under development, in addition to the need for care, functional needs also determined the likelihood of greater or lesser independence within the community living. Living homebound, a situation that has been associated with a higher risk of mortality (43, 44) and a reduced ability to perform instrumental activities outside the home (44), seemed to respond mainly to factors associated with mobility, with more than half of those living homebound being dependent for chair-to-bed transfers, eight out of ten unable to go up and down stairs independently, and nine out of ten unable to walk without assistance from others (30, 45). Bladder incontinence also played a role in the likelihood of being homebound. On the other hand, regarding the hypothesis that equilibrium plays a role in the observed results, Schirghuber et al. (46) noted that instability-equilibrium is a contributing factor in the outcome of living homebound.

The implication of mobility on personal independence within the home (25, 47) was reflected in social independence (26). Leaving the home, either independently or accompanied by another person, was usually limited by functional dependence in performing the activities of the Barthel index, particularly chair-to-bed transfers. People who were dependent on the Barthel index activities included in group 1 were also less likely to go for a walk or engage in leisure activities, and they also shopped independently less frequently. In these activities, urinary incontinence also played a role, affecting the ability to shop under supervision and not just independently.

Within the network associated with functional dependency, people with this type of dependency often need several types of support and assistance, ranging from mobility aids to caregivers assigned by social services or hired by the dependent individuals themselves. *A priori*, it seemed logical to assume that dependence on activities requiring mobility would be associated with a greater need to use mobility aids, to have assistants to help with domestic tasks and personal care, or to have an internal caregiver if these individuals were unable to care for themselves or move around efficiently.

Regarding the use of mobility aids, this aspect was confirmed (48, 49). Reduced mobility when chair-to-bed transfers or going up and down stairs meant greater functional limitations in terms of movement, and this was confirmed by the increased use of wheelchairs in these situations and, proportionally, less use of other mobility aids such as walkers or crutches-canes. This greater functional deterioration was also evident in the greater use of wheelchairs among people who were dependent on others for grooming, dressing-undressing, or toilet use, as well as a higher prevalence of fecal incontinence.

In addition, the use of a walker was more frequent in Barthel index activities in Group 1, that is, those associated with mobility, compared to what was observed in the rest of the activities. This result showed a gradient in the use of these devices to perform the activities included in Group 1, with greater use of wheelchairs, intermediate use of walkers, and less use of crutches-canes when dependent on others to perform the mobility-related activities included in this group.

Finally, regarding the use of crutches-canes, their use was more frequent among people who were independent in performing the Barthel index activity being evaluated, indicating that this mobility aid was used preferentially in the initial stages of functional decline.

In relation to the need for care, dependency often implies a greater need for care, which in our environment is usually provided by family members (24, 25, 29, 39, 43). However, on other occasions, it is provided by distinct types of assistants in the dependent person’s home. In this study, unlike what was observed about mobility aids, greater functional dependence in activities where mobility was a relevant factor was not associated with greater availability of public assistants to help with domestic tasks or personal care (50). This result indicates that other factors participate in the assignment of this aid (27).

When analyzing the possible reasons for not being assigned a public caregiver, care provided by family members may have filled this gap. As Li et al. (24) also note, the altruism of family members tends to compensate for social shortcomings. These caregivers, as noted in the studies by Martín et al. (42) and Giesbrecht et al. (51), are generally daughters, a situation that could reinforce the traditional female caregiving role and perpetuate social inequalities; it could also lead to personal stress due to overload, as suggested by the studies of Choy et al. (52) and Brandt et al. (53), and affect their finances and social participation, factors analyzed in the studies by Riffin et al. (27) and Martín et al. (42). All these factors could diminish the quality of life of these caregivers and, directly and indirectly, that of their environment, including the dependent person. The presence of caregivers from outside the family would reduce the burden, as noted by Choy et al. (52), with the availability of these resources from social institutions being a key factor.

On the other hand, having a private caregiver also showed that mobility was not a determining factor in their hiring. Among the justifications for this hiring in this study, dependence on activities included in Group 3, that is, those related to personal care in which mobility did not play a role, were shown to be the main reason. Lack of bladder and bowel control and needing help with feeding, along with other activities that, being in Group 2 because they required mobility to a greater or lesser extent, also fell under the pattern of personal care, such as dressing-undressing, or bathing, determined the need to have a privately hired support person. That is, it was the factors associated with the personal care environment itself that justified hiring a private caregiver.

Similarly, hiring an internal caregiver was favored when these personal care factors were present, also involving dependence on other activities, such as toilet use or grooming. Mobility, understood as an outward projection, to maintain social independence, was not as relevant as maintaining personal independence within the home. And, as with hiring a private caregiver, it was not shown to be a predominant factor in hiring an internal caregiver, but rather the main factor continued to be personal care.

Regarding the hiring of assistants to care for the dependent person, Orcasitas is a socioeconomically disadvantaged neighborhood in the city of Madrid. In this cohort, most had no education and almost half had low economic resources. Despite this, they made the financial effort to hire a private caregiver or resident caregiver. Doing so while limiting their personal finances for other situations, including essential living costs, meant that there was a level of dependency that justified this attitude. A level of dependency that was not supported, or not sufficiently according to the criteria of family members or the dependent persons themselves, by public provision of this assistance, raising the need for this outcome to be analyzed from a social and health planning perspective to avoid inequalities (50).

In summary, although the evolutionary path of functional dependence usually involves a progressive loss of functional abilities, this does not always occur, with stabilization at a certain level of deterioration being common (30). Added to this is the deterioration in other personal aspects associated with age, both of which contribute to a loss of abilities that can end up affecting personal and social independence in various areas (13, 25, 32, 33, 39, 54, 55). Considering these aspects in a changing society, such as today’s, it becomes a fundamental strategy, not only for the design of population studies, but also for health and social planning. Knowledge of the networks that are created around functional dependence would allow specific strategies to be designed for each nod-population group (30).

Starting from the interrelationships between these nodes, groups can be created that share specific characteristics. Groups in which the housing situation and community living conditions provide relevant information beyond that referred to by the level of functional dependence. In this sense, maintaining social independence required being able to leave the house, and being dependent for any activity on the Barthel index implied in this study a low probability of being able to leave the house independently (34). Furthermore, when this activity was conducted, it was mostly done accompanied by another person, only half of these people did so for walks or leisure activities, and less than one in ten of them were able to go shopping independently or under supervision during these outings. This shows the impact of functional dependence on social independence.

Regarding personal independence, the impact on autonomy of being dependent on others for feeding, toilet use, chair-to-bed transfers, grooming, or fecal incontinence was reflected in this study in the need to live with other people (13). At the same time, limiting one’s living space to one’s own home, i.e., living homebound, was favored by limited mobility, expressed through dependence on others for chair-to-bed transfers and going up and down stairs, as well as urinary incontinence. The provision of incontinence pads is funded by the public system for these people, but a certain social stigma prevails, limiting their use, with this result providing a new target for action by the healthcare system.

At the same time, maintaining personal independence could be facilitated using mobility aids, and in this study, the use of these devices helped to create profiles that allowed their use to be associated with specific characteristics of these people. In this regard, using a wheelchair was associated with a higher level of dependence for that activity, especially in activities in groups 1 and 2, while the use of crutches-cane was more frequent when individuals were independent for those activities. Only the activity of bathing was not associated with the use of any mobility aid.

On the other hand, when this personal independence was affected by a greater level of functional impairment, this situation meant a greater likelihood of having assistants in the home to provide personal care and perform domestic tasks. The results of this study suggest that these caregivers were probably hired in situations of advanced dependency, where mobility was no longer as relevant, but personal care was.

Finally, a significant percentage of people who lacked bladder and bowel control lived with their children or other people and often did not leave their homes. When they did, another person always accompanied them.

Analysis of the interrelationships between Barthel index activities allowed us to understand more about the reasons behind the conflicting results, establishing the key role of codependency and the type of codependency in the outcome (32, 33, 38). Results in which loss of mobility also played a role (47, 56). Understanding the extensive network that surrounds functional dependency will contribute to improving care for these individuals (3) and to adequate social and health planning (7, 26).

This point is particularly relevant in an entity, functional dependence in performing basic activities of daily living, which has a high mortality rate, as observed in this study. Except for urinary incontinence, all other Barthel index activities behaved as risk factors for mortality. However, the percentage of survival variance they explained was low, indicating that other factors participate in this mortality and should be analyzed in future studies.

These findings have several implications for clinical practice. First, geriatric assessments should pay special attention not only to the Barthel index score but also to the level of dependence for each specific activity within that index. And, in particular, two aspects of the functional assessment: (1) detecting incipient changes in the functional abilities of the activities included in Group 2 of this study, the onset of which may go unnoticed and which could be the first warning sign of the onset of functional decline. (2) Analyzing the level of interdependence between toilet use and bathing; this pairing could serve as an early indicator, within a hypothetical framework, of impaired balance and/or muscle weakness-loss of strength.

Both of these points are susceptible to specific interventions that can reverse or slow their progression, and could contribute, with minimal intervention, to healthy aging in older adults.

Lastly, the level of dependency for each of the Barthel index activities and the interrelationships between this level of dependency and the factors analyzed had a different effect on five-year survival. These differences could have implications in population studies, for example, those that assess intrinsic capacity, which tend to base survival analyses exclusively on the ability to walk independently for a certain distance. This criterion, which may be useful in people without functional dependence or with mild to moderate dependence, may not be useful for assessing mortality risk in people with severe dependence for ADL.

The Barthel index was not developed as a test to assess mortality risk, but rather to estimate functional capacity (1); however, it has been used for this purpose in numerous studies (11, 12, 57–59). Analyses using ROC curves and the Delong method established in this study that its utility as a test could be classified as poor, as it was below 0.7, the threshold used as a criterion for acceptable performance. This result implied that the Barthel index had low discriminatory power in this cohort as a prognostic marker of mortality or in identifying mortality risk groups. It was in contrast to the findings of other studies, such as those by Torres Moreno et al. (60) and Bretos-Azcona et al. (61), where performance was acceptable.

On the other hand, when analyzing each activity of the Barthel index specifically, the results were similar, except for two activities related to bowel and bladder control, for which the probability was close to that of random chance. This result raises a question, within this hypothesis-generating study, regarding the actual utility of including bowel and bladder control in a tool that, although designed to assess functional capacity, is currently used in various settings to estimate the probability of survival associated with functional capacity (60, 61). For the remaining activities included in the Barthel index, the performance of the ROC curves showed limited capacity, with a 61–67% probability of correctly classifying a random pair consisting of one case that dies and another that does not.

Although these overall results suggest that the clinical utility of both the Barthel index and each activity individually was not significant in this cohort, this finding must be put into context. The advanced age of this cohort may have contributed to the observed result. It may also be due to the fact that the study compared a population with severe and moderate functional dependence, rather than a functionally dependent population versus a non-dependent population, as is typically done in population-based studies (60, 61). In any case, the clinical utility of the Barthel index at the population level is unquestionable, as supported by numerous studies, and it constitutes a fundamental tool in addressing vulnerable populations (62).

The main limitations of this study are, first, the small sample size. Although this sample represents the entire population with functional dependence in a neighborhood of Madrid, its size reduces the statistical power of the analysis of subgroups, limiting the generalizability of the observed results.

Second, a potentially lower “cognitive reserve,” secondary to a lower educational level and lower socioeconomic status, may have influenced the observed levels of functional dependence and the interrelationships between these levels and the personal and social environment, which could differ in other populations with distinct socioeconomic characteristics (63).

Furthermore, thirdly, although low economic and cultural status may have influenced the fact that, in this cohort, hiring private or internal caregivers was a determining factor in personal care, it cannot be ruled out that, in other settings, particularly those that are socioeconomically advantaged, other priorities, such as maintaining a social life outside the home, may influence these hiring criteria (64, 65).

Fourth, regarding other factors influencing the presence of functional dependence, the mean age of this cohort was high; and, considering the five-year follow-up period, this mean would have reached 91 years had no participants died. In this age group, age-related mortality played a role that may have influenced the results. These results might also differ in other groups of younger dependent individuals (66). Within this same factor, age, and as is the case in most population-based studies conducted with older adults, the male population was underrepresented due to their lower life expectancy; therefore, the results observed in this study should be confirmed in studies with larger cohorts of men. In addition to age and sex, other factors may have influenced the survival analysis, such as the burden of disease, which was not included in the analyzed model.

Finally, the classification of activities in the Barthel index used in this study was based on operational considerations, employing subjective criteria derived from observations of daily activities in the care of this population group; this classification requires validation in other studies involving larger groups of individuals with functional dependence.

## Conclusion

Functional dependence for performing basic activities of daily living establishes a complex network of interrelationships that affect the personal and social independence of the dependent person and shape their way of life in the community. Understanding these networks is vitally important for promoting healthy aging and enabling the population to remain in the community.

This study found that mobility played a significant role in maintaining social independence, particularly in the ability to leave the home and engage in activities outside the home, but this role was shared when the need to live with children was evaluated, and ended up being residual when the criteria by which these individuals ended up needing a private caregiver or internal caregiver were analyzed.

As expected, the use of mobility assistance devices responded to mobility criteria, but the use of these devices was also associated with the loss of the ability to perform those activities that transform us from a status of homebound person to a person who still maintains the ability or hope of going further, such as the ability to dressing-undressing, or grooming, and take the next step of leaving the house.

## Data Availability

All data necessary for the proper evaluation and interpretation of the study have been included in the manuscript or provided as Supplementary material in the Appendices.
