# Supplementary material for: Association between the different basic activities of daily living on the Barthel index and community living, use of mobility aids, and the survival at 5 years
Source: Front Public Health. 2026 Jun 4;14:1825340. doi: 10.3389/fpubh.2026.1825340 (PMC13277337; doi:10.3389/fpubh.2026.1825340)
Supplement: Supplementary file 1 [file Supplementary_File_1.pdf]

Appendix 1: Tables A1, A2, A3, A4, A5, A6, A7, A8 and A9

Table A1: Interrelationships between the level of dependency for each activity in the Barthel index and the sociodemographic indicators sex, economic status, and educational level. Cell color format: Mustard color: group 1 activities of the Barthel index. Green color: group 2 activities of the Barthel index. Blue color: group 3 activities of the Barthel index.

Barthel Index activities and sociodemographic and socioeconomic data of the functionally dependent population of Orcasitas										
Barthel Index Activity	Activity	Sex Level		Statistical test	Economic level Level		Statistical test	Education level Level		Statistical test
	Level	Female	Male	Odds ratio (Confidence interval)	<11.200 euros/year	>11.200 euros/year	Odds ratio (Confidence interval)	No education	Has formal education	Odds ratio (Confidence interval)
**Chair-to-bed transfers**	**Dependent**	41% (41)	44.4% (12)	0.869 (0.369-2.047)	45% (27)	38.8% (26)	1.290 (0.636-2.617)	38.7% (43)	60% (9)	0.422 (0.140-1.268)
	**Independent**	59% (59)	55.6% (15)		55% (33)	61.2% (41)		61.3% (68)	40% (6)	
**Up-down stairs**	**Dependent**	61% (61)	66.7% (18)	0.782 (0.319-1.915)	68.3% (41)	56.7% (38)	1.647 (0.796-3.409)	59.5% (66)	80% (12)	0.367 (0.098-1.374)
	**Independent**	39% (39)	33.3% (19)		31.7% (19)	43.3% (29)		40.5% (45)	20% (3)	
**Mobility on level surfaces**	**Dependent**	21% (21)	18.5% (5)	1.170 (0.396-3.457)	28.3% (17)	13.4% (9)	**2.548 (1.037-6.261)**	18.9% (21)	26.7% (4)	0.642 (0.186-2.215)
	**Independent**	79% (79)	81.5% (22)		71.7% (43)	86.6% (58)		81.1% (90)	73.35 (11)	
**Toilet use**	**Dependent**	18% (18)	18.5% (5)	0.966 (0.323-2.893)	20% (12)	16.4% (11)	1.273 (0.515-3.144)	17.1% (19)	26.7% (4)	0.568 (0.163-1.975)
	**Independent**	82% (82)	81.5% (22)		80% (48)	83.6% (56)		82.9% (92)	73.3% (11)	
**Dressing-undressing**	**Dependent**	29% (29)	40.7% (11)	0.594 (0.246-1.434)	35% (21)	28.4% (19)	1.360 (0.642-2.882)	27.9% (31)	53.3% (8)	0.339 (0.113-1.014)
	**Independent**	71% (71)	59.3% (16)		65% (39)	71.6% (48)		72.1% (80)	46.7% (7)	
**Grooming**	**Dependent**	49% (49)	44.4% (12)	1.201 (0.511-2.822)	50% (30)	46.3% (31)	1.161 (0.578-2.333)	45% (50)	66.7% (10)	0.410 (0.131-1.277)
	**Independent**	51% (51)	55.6% (15)		50% (30)	53.75 (36)		55% (61)	33.35 (5)	
**Bathing**	**Dependent**	75% (75)	63% (17)	1.765 (0.715-4.353)	73.3% (44)	71.6% (48)	1.089 (0.499-2.376)	69.4% (77)	93.3% (14)	0.162 (0.020-1.280)
	**Independent**	25% (25)	37% (10)		26.7% (16)	28.45 (19)		30.6% (34)	6.7% (1)	
**Feeding**	**Dependent**	31% (31)	25.9% (7)	1.284 (0.492-3.350)	35% (21)	25.4% (17)	1.584 (0.737-3.401)	27% (30)	46.7% (7)	0.423 (0.141-1.268)
	**Independent**	69% (69)	74.1% (20)		65% (39)	74.6% (50)		73% (81)	53.3% (8)	
**Bladder**	**Dependent**	35% (35)	33.3% (9)	1.077 (0.438-2.647)	36.7% (22)	32.8% (22)	1.184 (0.570-2.462)	33.3% (37)	40% (6)	0.750 (0.248-2.266)
	**Independent**	65% (65)	66.7% (18)		63.3% (38)	67.2% (45)		66.7% (74)	60% (9)	
**Bowels**	**Dependent**	20% (20)	22.2% (6)	0.875 (0.312-2.454)	16.7% (10)	23.9% (16)	0.638 (0.264-1.539)	21.6% (24)	13.3% (2)	1.793 (0.378-8.497)
	**Independent**	80% (80)	77.8% (21)		83.3% (50)	76.1% (51)		78.4% (87)	86.7% (13)	

Table A2: Analysis of the relationship between the level of dependence in performing each of the activities in the Barthel index and personal and social independence in the community, defined by housing situation (lives independently versus lives with children or other people) and community living arrangements (lives confined versus can leave home to go for walks or engage in leisure activities). Cell color format: Mustard color: group 1 activities of the Barthel index. Green color: group 2 activities of the Barthel index. Blue color: group 3 activities of the Barthel index.

**Barthel Index: personal and social independence**								
**Barthel Index Activity**	**Activity**	**Housing situation**		**Statistical test**	**Leaving home**		**Statistical test**
	**Level**	**Lives independently**	**lives with other people**	**Odds ratio (Confidence interval)**	**Homebound**	**He leaves his house**	**Odds ratio (Confidence interval)**
**Chair-to-bed transfers**	**Dependent**	18.9% (10)	81.1% (43)	**4.300 (1.883-9.815)**	52.8% (28)	47.2% (25)	**0.266 (0.124-0.572)**
	**Independent**	50% (37)	50% (37)		23% (17)	77% (57)	
**Up-down stairs**	**Dependent**	34.2% (27)	65.8% (52)	1.375 (0.657-2.879)	45.6% (36)	54.4% (43)	**0.276 (0.118-0.644)**
	**Independent**	41.7% (20)	58.3% (28)		18.8% (9)	81.3% (39)	
**Mobility on level surfaces**	**Dependent**	21.3% (10)	78.7% (37)	0.354 (0.124-1.007)	38.2% (42)	61.8% (68)	0.346 (0.094-1.279)
	**Independent**	8.8% (7)	91.3% (73)		17.6% (3)	82.4% (14)	
**Toilet use**	**Dependent**	13% (3)	87% (20)	**4.888 (1.367-17.484)**	47.8% (11)	52.2% (12)	0.530 (0.212-1.323)
	**Independent**	42.3% (44)	57.7% (60)		32.7% (34)	67.3% (70)	
**Dressing-undressing**	**Dependent**	20% (8)	80% (32)	**3.250 (1.344-7.854)**	52.5% (21)	47.5% (19)	0.471 (0.218-1.019)
	**Independent**	44.8% (39)	55.2% (48)		29.9% (26)	70.1% (61)	
**Grooming**	**Dependent**	34% (16)	56.3% (45)	**2.491 (1.179-5.261)**	42.6% (26)	57.4% (35)	0.544 (0.261-1.136)
	**Independent**	66% (31)	43.8% (35)		28.8% (19)	71.2% (47)	
**Bathing**	**Dependent**	34.8% (32)	65.2% (60)	1.406 (0.634-3.114)	37% (34)	63% (58)	0.782 (0.341-1.793)
	**Independent**	42.9% (15)	57.1% (20)		31.4% (11)	68.6% (24)	
**Feeding**	**Dependent**	10.5% (4)	89.5% (34)	**7.945 (2.602-24.263)**	39.5% (15)	60.5% (23)	0.780 (0.356-1.709)
	**Independent**	48.3% (43)	51.7% (46)		33.7% (30)	66.3% (59)	
**Bladder**	**Dependent**	29.5% (13)	70.5% (31)	1.654 (0.757-3.615)	47.7% (21)	52.3% (23)	**0.446 (0.209-0.951)**
	**Independent**	41% (34)	59% (49)		28.9% (24)	71.1% (59)	
**Bowels**	**Dependent**	15.4% (4)	84.6% (22)	**4.077 (1.309-12.699)**	46.2% (12)	53.8% (14)	0.566 (0.236-1.360)
	**Independent**	42.6% (43)	57.4% (58)		32.7% (33)	67.3% (68)	

Table A3: Analysis of the relationship between the level of dependence in performing each of the activities in the Barthel index and the mode of community living, defined by the ability to leave the home versus living homebound, and, within the ability to leave the home, whether to do so independently or accompanied by another person. Cell color format: Mustard color: group 1 activities of the Barthel index. Green color: group 2 activities of the Barthel index. Blue color: group 3 activities of the Barthel index.

**Barthel index and relationship with the outside world**					
**Variable**		**Relationship with the outside world**			
**Barthel index activity**	**Level**	**Homebound**	**Leaves home independently**	**Leaves home with another person**	**Chi-square (p)**
**Chair-to-bed transfers**	Dependent	50.9% (27)	1,9% (1)	47.2% (25)	**χ^2= 16.816 (p<0.001)**
	Independent	21.6% (16)	20.3% (15)	58.1% (43)	
**Up-down stairs**	Dependent	44.3% (35)	2.5% (2)	53.2% (42)	**χ^2= 23.555 (p<0.001)**
	Independent	16.7% (8)	29.2% (14)	54.2% (26)	
**Mobility on level surfaces**	Dependent	50% (13)	0% (0)	50% (13)	**χ^2= 6.711 (p=0.035)**
	Independent	29.7% (30)	15.8% (16)	54.5% (55)	
**Toilet use**	Dependent	47.8% (11)	0% (0)	52.2% (12)	χ^2= 5.167 (p=0.076)
	Independent	30.8% (32)	15.4% (16)	53.8% (56)	
**Dressing-undressing**	Dependent	47.5% (19)	2.5% (19)	50% (20)	**χ^2= 8.073 (p=0.018)**
	Independent	27.6% (24)	17.2% (15)	55.2% (48)	
**Grooming**	Dependent	41% (25)	3.3% (2)	55.7% (34)	**χ^2= 9.958 (p=0.007)**
	Independent	27.3% (18)	21.2% (14)	51.5% (34)	
**Bathing**	Dependent	34.8% (32)	4.3% (4)	60.9% (56)	**χ^2= 21.468 (p<0.001)**
	Independent	31.4% (11)	34.3% (12)	34.3% (12)	
**Feeding**	Dependent	39.5% (15)	0% (0)	60.5% (23)	**χ^2= 7.830 (p=0.020**
	Independent	31.5% (28)	18% (16)	50.6% (45)	
**Bladder**	Dependent	45.5% (20)	0% (0)	54.5% (24)	**χ^2= 11.168 (p=0.004)**
	Independent	27.7% (23)	19.3% (16)	53% (44)	
**Bowels**	Dependent	46.2% (12)	0% (0)	53.8% (14)	χ^2= 5.579 (p=0.061)
	Independent	30.7% (31)	15.8% (16)	53.5% (54)	

Table A4: Instrumental activities and ability to perform basic activities of daily living. Interrelationships between the level of functional dependence for performing each of the activities in the Barthel index and the instrumental activities of leaving the house for a walk or leisure, shopping independently, and shopping under supervision. Cell color format: Mustard color: group 1 activities of the Barthel index. Green color: group 2 activities of the Barthel index. Blue color: group 3 activities of the Barthel index.

Barthel Index: activities associated with personal independence										
Barthel Index Activity	Activity	Leaves home for walks or leisure activities		Statistical test	Shops independently		Statistical test	Shops under supervision		Statistical test
		Level			Level			Level		
	Level	Yes	No	Odds ratio (Confidence interval)	Yes	No	Odds ratio (Confidence interval)	Yes	No	Odds ratio (Confidence interval)
**Chair-to-bed transfers**	**Dependent**	40.8% (20)	59.2% (29)	**0.265 (0.123-0.572)**	4% (2)	96% (48)	**0.137 (0.030-0.624)**	6.1% (3)	93.9% (46)	0.275 (0.075-1.014)
	**Independent**	72.2% (52)	27.8% (20)		23.3% (17)	76.7% (56)		19.2% (14)	80.8% (59)	
**Up-down stairs**	**Dependent**	50% (37)	50% (37)	**0.343 (0.154-0.762)**	5.3% (4)	94.7% (71)	**0.124 (0.038-0.402)**	9.5% (7)	90.5% (67)	0.397 (0.140-1.129)
	**Independent**	74.5% (35)	25.5% (12)		31.3% (15)	68.8% (33)		20.8% (10)	79.2% (38)	
**Mobility on level surfaces**	**Dependent**	39.1% (9)	60.9% (14)	**0.357 (0.140-0.909)**	8.3% (2)	91.7% (22)	0.439 (0.094-2.043)	8.7% (2)	91.3% (21)	0.533 (0.113-2.515)
	**Independent**	64.3% (63)	35.7% (35)		17.2% (17)	82.8% (82)		15.2% (15)	84.8% (84)	
**Toilet use**	**Dependent**	47.6% (10)	52.4% (11)	0.557 (0.216-1.436)	9.1% (2)	90.9% (20)	0.494 (0.105-2.315)	**0% (0)**	**100% (21)**	---
	**Independent**	62% (62)	38% (38)		16.8% (17)	83.2% (84)		**16.8% (17)**	**83.2% (84)**	
**Dressing-undressing**	**Dependent**	47.2% (17)	52.8% (19)	0.488 (0.221-1.077)	5.4% (2)	94.6% (35)	0.232 (0.051-1.061)	5.6% (2)	94.4% (34)	0.278 (0.060-1.287)
	**Independent**	64.7% (55)	35.3% (30)		19.8% (17)	80.2% (69)		17.4% (15)	82.6% (71)	
**Grooming**	**Dependent**	50% (28)	50% (28)	**0.477 (0.228-0.998)**	8.8% (5)	91.2% (52)	0.357 (0.120-1.063)	8.9% (5)	91.1% (51)	0.441 (0.145-1.340)
	**Independent**	67.7% (44)	32.3% (21)		21.2% (14)	78.8% (53)		18.2% (12)	81.8% (54)	
**Bathing**	**Dependent**	50% (17)	50% (17)	0.582 (0.261-1.296)	8% (7)	92% (81)	**0.166 (0.058-0.469)**	9.2% (8)	90.8% (79)	**0.293 (0.102-0.836)**
	**Independent**	63.2% (55)	36.8% (32)		34.3% (12)	65.7% (23)		25.7% (9)	74.35 (26)	
**Feeding**	**Dependent**	58.1% (50)	41.9% (36)	0.821 (0.366-1.842)	5.7% (2)	94.3% (33)	0.253 (0.055-1.160)	8.8% (3)	91.2% (31)	0.512 (0.137-1.906)
	**Independent**	62.9% (22)	37.1% (13)		19.3% (17)	80.7% (71)		15.9% (14)	84.1% (74)	
**Bladder**	**Dependent**	46.3% (19)	53.7% (22)	**0.440 (0.204-0.949)**	4.8% (2)	95.2% (40)	**0.188 (0.041-0.858)**	2.4% (1)	97.6% (40)	**0.102 (0.013-0.796)**
	**Independent**	66.3% (53)	33.8% (27)		21% (17)	79% (64)		19.8% (16)	80.2% (65)	
**Bowels**	**Dependent**	54.2% (13)	45.8% (11)	0.761 (0.309-1.873)	8% (2)	92% (23)	0.414 (0.089-1.926)	**0% (0)**	**100% (24)**	---
	**Independent**	60.8% (59)	39.2% (38)		17.3% (23)	82.7% (81)		**17.3% (17)**	**82.7% (81)**	

Table A5: Analysis of the relationship between the level of dependence in performing each of the Barthel index activities and the use of mobility assistance devices. Cell color format: Mustard color: group 1 activities of the Barthel index. Green color: group 2 activities of the Barthel index. Blue color: group 3 activities of the Barthel index.

Barthel Index Activities and Use of Assistance Devices for Mobility										
Barthel Index Activity	Activity	Wheelchair		Statistical test	Walker		Statistical test	Crutches-cane		Statistical test
	Level	Yes	No	Odds ratio (Confidence interval)	Yes	No	Odds ratio (Confidence interval)	Yes	No	Odds ratio (Confidence interval)
Chair-to-bed transfers	Dependent	49.1% (26)	50.9% (27)	**3.788 (1.733-8.278)**	37.7% (20)	62.3% (33)	1.188 (0.569-2.478)	20.8% (11)	79.2% (42)	**0.189 (0.084-0.424)**
	Independent	20.3% (15)	79.7% (59)		33.8% (25)	66.2% (49)		58.1% (43)	41.9% (31)	
Up-down stairs	Dependent	43% (34)	57% (45)	**4.425 (1.769-11.071)**	39.2% (31)	60.8% (48)	1.568 (0.727-3.384)	35.4% (28)	64.6% (51)	**0.465 (0.224-0.965)**
	Independent	14.6% (7)	85.4% (41)		29.2% (14)	70.8% (34)		54.2% (26)	45.8% (22)	
Mobility on level surfaces	Dependent	34.5% (38)	65.5% (72)	2.882 (0.782-10.628)	36.4% (40)	63.6% (70)	0.729 (0.240-2.220)	43.6% (48)	56.4% (62)	0.705 (0.243-2.041)
	Independent	17.6% (3)	82.4% (14)		29.4% (5)	70.6% (12)		35.3% (6)	64.7% (11)	
Toilet use	Dependent	52.2% (12)	47.8% (11)	**2.821 (1.120-7.105)**	26.1% (6)	73.9% (17)	0.588 (0.214-1.168)	17.4% (4)	82.6% (19)	**0.227 (0.072-0.714)**
	Independent	27.9% (29)	72.1% (75)		37.5% (39)	62.5% (65)		48.1% (50)	51.9% (54)	
Dressing-undressing	Dependent	52.5% (21)	47.5% (19)	**3.703 (1.669-8.212)**	27.5% (11)	72.5% (29)	0.591 (0.261-1.338)	22.5% (9)	77.5% (31)	**0.271 (0.116-0.636)**
	Independent	23% (20)	77% (67)		39.1% (34)	60.9% (53)		51.7% (45)	48.3% (42)	
Grooming	Dependent	44.3% (27)	55.7% (34)	**2.950 (1.356-6.414)**	34.4% (21)	65.6% (40)	0.919 (0.443-1.903)	31.1% (19)	68.9% (42)	**0.401 (0.194-0.828)**
	Independent	21.2% (14)	78.8% (52)		36.4% (24)	63.6% (42)		53% (35)	47% (31)	
Bathing	Dependent	35.9% (33)	64.1% (59)	1.888 (0.770-4.627)	34.8% (32)	65.2% (60)	0.903 (0.402-2.026)	38% (35)	62% (57)	0.517 (0.235-1.136)
	Independent	22.9% (8)	77.1% (27)		37.1% (13)	62.9% (22)		54.3% (19)	45.7% (16)	
Feeding	Dependent	39.5% (15)	60.5% (23)	1.580 (0.714-3.499)	28.9% (11)	71.1% (27)	0.659 (0.290-1.498)	28.9% (11)	71.1% (27)	**0.436 (0.193-0.985)**
	Independent	29.2% (26)	70.8% (63)		38.2% (34)	61.8% (55)		48.3% (43)	51.7% (46)	
Bladder	Dependent	43.2% (19)	56.8% (25)	2.107 (0.975-4.553)	29.5% (13)	70.5% (31)	0.668 (0.305-1.464)	27.3% (12)	72.7% (32)	**0.366 (0.166-0.807)**
	Independent	26.5% (22)	73.5% (61)		38.6% (32)	61.4% (51)		50.6% (42)	49.4% (41)	
Bowels	Dependent	50% (13)	50% (13)	**2.607 (1.077-6.308)**	23.1% (6)	76.9% (20)	0.477 (0.176-1.292)	30.8% (8)	69.2% (18)	0.531 (0.212-1.334)
	Independent	27.7% (28)	72.3% (73)		38.6% (39)	61.4% (62)		45.5% (46)	54.5% (55)	

Table A6: Analysis of the relationship between the level of dependence for performing each of the Barthel index activities and the availability of assistants for domestic tasks and personal care, or of an internal caregiver. Cell color format: Mustard color: group 1 activities of the Barthel index. Green color: group 2 activities of the Barthel index. Blue color: group 3 activities of the Barthel index.

Barthel Index activities and availability of caregivers										
**Barthel Index Activity**	**Activity**	**Public caregiver**		**Statistical test**	**Private caregiver**		**Statistical test**	**Internal caregiver**		**Statistical test**
	**Level**	**Yes**	**No**	**Odds ratio (Confidence interval)**	**Yes**	**No**	**Odds ratio (Confidence interval)**	**Yes**	**No**	**Odds ratio (Confidence interval)**
**Chair-to-bed transfers**	**Dependent**	50.9% (27)	49.1% (26)	0.725 (0.355-1.477)	39.6% (21)	60.4% (32)	1.285 (0.614-2.688)	22.6% (12)	77.4% (41)	1.463 (0.599-3.575)
	**Independent**	58.9% (43)	41.1% (30)		33.8% (24)	66.2% (47)		16.7% (12)	83.3% (60)	
**Up-down stairs**	**Dependent**	58.2% (46)	41.8% (33)	1.336 (0.646-2.761)	41.8% (33)	58.2% (46)	1.973 (0.888-4.381)	17.7% (14)	82.3% (65)	0.775 (0.313-1.922)
	**Independent**	51.1% (24)	48.9% (23)		26.7% (12)	73.3% (33)		21.7% (10)	78.3% (36)	
**Mobility on level surfaces**	**Dependent**	57.3% (63)	42.7% (47)	0.580 (0.202-1.671)	39.8% (43)	60.2% (65)	**0.216 (0.047-0.998)**	20.9% (23)	79.1% (87)	0.270 (0.034-2.163)
	**Independent**	43.8% (7)	56.3% (9)		12.5% (2)	87.5% (14)		6.7% (1)	93.3% (14)	
**Toilet use**	**Dependent**	60.9% (14)	39.1% (9)	1.306 (0.519-3.285)	52.2% (12)	47.8% (11)	2.248 (0.898-5.628)	39.1% (9)	60.9% (14)	**3.729 (1.371-10.143)**
	**Independent**	54.4% (56)	45.6% (47)		32.7% (33)	67.3% (68)		14.7% (15)	85.3% (87)	
**Dressing-undressing**	**Dependent**	55% (22)	45% (18)	0.968 (0.455-2.057)	50% (20)	50% (20)	**2.360 (1.086-5.130)**	30% (12)	70% (28)	**2.607 (1.048-6.484)**
	**Independent**	55.8% (48)	44.2% (38)		29.8% (25)	70.2% (59)		14.1% (12)	85.9% (73)	
**Grooming**	**Dependent**	52.5% (32)	47.5% (29)	0.784 (0.388-1.585)	41.7% (25)	58.3% (35)	1.571 (0.752-3.283)	29.5% (18)	70.5% (43)	**4.047 (1.482-11.050)**
	**Independent**	58.5% (38)	41.5% (27)		31.3% (20)	68.8% (44)		9.4% (6)	90.6% (58)	
**Bathing**	**Dependent**	57.1% (52)	42.9% (39)	1.259 (0.576-2.753)	44.9% (40)	55.1% (49)	**4.898 (1.740-13.785)**	24.2% (22)	75.8% (69)	**5.101 (1.130-23.024)**
	**Independent**	51.4% (18)	48.6% (17)		14.3% (5)	85.7% (30)		5.9% (2)	94.1% (32)	
**Feeding**	**Dependent**	47.4% (18)	52.6% (20)	0.623 (0.290-1.340)	57.9% (22)	42.1% (16)	**3.766 (1.689-8.396)**	34.2% (13)	65.8% (25)	**3.593 (1.430-9.028)**
	**Independent**	59.1% (52)	40.9% (36)		26.7% (23)	73.3% (63)		12.6% (11)	87.4% (76)	
**Bladder**	**Dependent**	54.5% (24)	45.5% (20)	0.939 (0.450-1.961)	50% (22)	50% (22)	**2.478 (1.154-5.320)**	27.3% (12)	72.7% (32)	2.156 (0.874-5.321)
	**Independent**	56.1% (46)	43.9% (36)		28.8% (23)	71.3% (57)		14.8% (12)	85.2% (69)	
**Bowels**	**Dependent**	53.8% (14)	46.2% (12)	0.917 (0.385-2.180)	53.8% (14)	46.2% (12)	**2.522 (1.045-6.084)**	26.9% (7)	73.1% (19)	1.777 (0.646-4.888)
	**Independent**	56% (56)	44% (44)		31.6% (31)	68.4% (67)		17.2% (17)	82.9% (82)	

Table A7: Analysis of the relationship between the level of dependency for each Barthel Index activity and survival at five years of follow-up. Cell color format: Mustard color: group 1 activities of the Barthel index. Green color: group 2 activities of the Barthel index. Blue color: group 3 activities of the Barthel index.

Barthel Index activities and five-years survival				
**Barthel Index Activity**	**Survival**			**Statistical test**
	**Level**	**Alive**	**Deceased**	**Odds ratio (Confidence interval)**
**Chair-to-bed transfers**	**Dependent**	20.8% (11)	79.2% (42)	**0.248 (0.111-0.555)**
	**Independent**	51.4% (38)	48.6% (36)	
**Up-down stairs**	**Dependent**	26.6% (21)	73.4% (58)	**0.259 (0.121-0.553)**
	**Independent**	58.3% (28)	41.7% (20)	
**Mobility on level surfaces**	**Dependent**	32.7% (36)	67.3% (74)	**0.149 (0.045-0-491)**
	**Independent**	76.5% (13)	23.5% (4)	
**Toilet use**	**Dependent**	8.7% (2)	91.3% (21)	**0.116 (0.026-0.518)**
	**Independent**	45.2% (47)	54.8% (57)	
**Dressing-undressing**	**Dependent**	12.5% (5)	87.5% (35)	**0.140 (0.050-0.390)**
	**Independent**	50.6% (44)	49.4% (43)	
**Grooming**	**Dependent**	26.2% (16)	73.8% (45)	**0.356 (0.168-0.751)**
	**Independent**	50% (33)	50% (33)	
**Bathing**	**Dependent**	30.4% (28)	69.6% (64)	**0.292 (0.130-0.655)**
	**Independent**	60% (21)	40% (14)	
**Feeding**	**Dependent**	21.1% (8)	78.9% (30)	**0.312 (0.129-0.756)**
	**Independent**	46.1% (41)	53.9% (48)	
**Bladder**	**Dependent**	27.3% (12)	72.7% (32)	0.466 (0.211-1.029)
	**Independent**	44.6% (37)	55.4% (46)	
**Bowels**	**Dependent**	19.2% (5)	80.8% (21)	**0.308 (0.108-0.883)**
	**Independent**	43.6% (44)	56.4% (57)	

Table A8: Five-year survival rate based on the level of dependency for each group of activities. Data relating to being dependent for all activities included in each operational group and being dependent for at least one of the activities included in each group. Cell color format: Mustard color: group 1 activities of the Barthel index. Green color: group 2 activities of the Barthel index. Blue color: group 3 activities of the Barthel index.

**Barthel Index Activity Groups and 5-Year Survival Rates**				
**Dependent on all activities included in the group versus independent on all activities included in the group**				
**Barthel Index Activity Group**	**Survival**			**Statistical test**
	**Level**	**Alive**	**Deceased**	**Odds ratio (Confidence interval)**
**Group 1:<br>Mobility on level surfaces<br>Chair-to-bed transfers<br>Up-down stairs<br>Toilet use**	**Dependent**	0% (0)	100% (11)	—
	**Independent**	57.5% (23)	42.5% (17)	
**Group 2:<br>Dressing-undressing<br>Grooming<br>Bathing**	**Dependent**	8.3% (3)	91.7% (33)	**16.000 (3.908-65.493)**
	**Independent**	59.3% (16)	40.7% (11)	
**Group 3:<br>Feeding<br>Bladder<br>Bowels**	**Dependent**	14.3% (2)	85.7% (12)	**6.375 (1.322-30.731)**
	**Independent**	51.5% (34)	48.5% (32)	

Dependent on at least one activity included in the group versus independent in at least one activity				
Barthel Index Activity Group	Survival			Statistical test
	Level	Alive	Deceased	Odds ratio (Confidence interval)
Group 1:<br>Mobility on level surfaces<br>Chair-to-bed transfers<br>Up-down stairs<br>Toilet use	Dependent	29.9% (26)	70.1% (66)	1.856<br>(1.034-3.330)
	Independent	42.2% (49)	57.8% (67)	
Group 2:<br>Dressing-undressing<br>Grooming<br>Bathing	Dependent	33% (33)	67% (67)	2.075<br>(1.155-3.726)
	Independent	50.5% (46)	49.5% (45)	
Group 3:<br>Feeding<br>Bladder<br>Bowels	Dependent	24.6% (15)	75.4% (46)	2.183<br>(1.092-4.364)
	Independent	41.6% (47)	58.4% (66)	

Table A9: Linear regression analysis of survival variance for each Barthel index activity. Cell color format: Mustard color: group 1 activities of the Barthel index. Green color: group 2 activities of the Barthel index. Blue color: group 3 activities of the Barthel index.

Survival variance using linear regression for each Barthel index activity								
Activity	R	R^2	Adjusted R^2	SE	t	Sig.	CI 95,0% for B	
							Lower	Upper
**Chair-to-bed transfers**	0.310	0.096	0.089	0.467	-3.645	0.001	-0.472	-0.140
**Up-down stairs**	0.316	0.100	0.093	0.465	-3.727	0.001	-0.486	-0.149
**Mobility on level surfaces**	0.162	0.026	0.018	0.484	-1.831	0.069	-0.406	0.016
**Toilet use**	0.289	0.083	0.076	0.470	-3.372	0.001	-0.579	-0.151
**Dressing-undressing**	0.363	0.132	0.125	0.457	-4.360	0.001	-0.554	-0.208
**Grooming**	0.244	0.060	0.052	0.476	-2.813	0.006	-0.405	-0.070
**Bathing**	0.271	0.074	0.066	0.472	-3.152	0.002	-0.481	-0.110
**Feeding**	0.235	0.055	0.048	0.477	-2.707	0.008	-0.433	-0.067
**Bladder**	0.169	0.029	0.021	0.484	-1.919	0.057	-0.352	0.005
**Bowels**	0.202	0.041	0.033	0.481	-2.302	0.023	-0.453	-0.034
**All activities**	0.465	0.216	0.148	0.451	--	--	--	--
