# Supplementary material for: Association between the different basic activities of daily living on the Barthel index and community living, use of mobility aids, and the survival at 5 years
Source: Front Public Health. 2026 Jun 4;14:1825340. doi: 10.3389/fpubh.2026.1825340 (PMC13277337; doi:10.3389/fpubh.2026.1825340)
Supplement: Supplementary file 2 [file Supplementary_File_2.pdf]

Appendix 2: Tables S1, S2, and S3

Table S1: Group 1: Barthel index activities in which mobility is essential to their performance and are relevant to personal and social independence. Association between these activities and sociodemographic factors, community living, need for mobility aids and/or home caregivers. Impact of dependence on group 1 activities on survival. 1: lives independently (alone or with a partner) - lives with other people (children, people who are neither children nor partners)

Barthel Index: activities associated with mobility as a factor of personal and social independence													
**Social factor**	**Activity**	**Chair-to-bed transfers**		**Statistical test**	**Up-Down stairs**		**Statistical test**	**Mobility on level surfaces**		**Statistical test**	**Toilet use**		**Statistical test**
		**Level**			**Level**			**Level**			**Level**		
	**Level**	**Dependent or major help**	**Independent or minor help**	**Odds ratio (Confidence interval)**	**Dependent**	**Needs help or independent**	**Odds ratio (Confidence interval)**	**Not walking or dependent**	**Independent**	**Odds ratio (Confidence interval)**	**Dependent**	**Independent or minor help**	**Odds ratio (Confidence interval)**
**Housing situation**	**Lives independently**	18.9% (10)	50% (37)	**0.233 (0.102-0.531)**	34.2% (27)	41.7% (20)	0.727 (0.347-1.521)	21.3% (10)	8.8% (7)	2.819 (0.993-8.004)	13% (3)	42.3% (44)	**0.205 (0.057-0.732)**
	**lives with other people**	81.1% (43)	50% (37)		65.8% (52)	58.3% (28)		78.7% (37)	91.3% (73)		87% (20)	57.7% (60)	
**Leaving home**	**Homebound**	52.8% (28)	23% (17)	**0.266 (0.124-0.572)**	45.6% (36)	18.8% (9)	**0.276 (0.118-0.644)**	38.2% (42)	17.6% (3)	2.882 (0.782-10.628)	47.8% (11)	32.7% (34)	0.530 (0.212-1.323)
	**He leaves his house**	47.2% (25)	77% (57)		54.4% (43)	81.3% (39)		61.8% (68)	82.4% (14)		52.2% (12)	67.3% (70)	
**Wheelchair**	**Yes**	49.1% (26)	20.3% (15)	**3.788 (1.733-8.278)**	43% (34)	14.6% (7)	**4.425 (1.769-11.071)**	34.5% (38)	17.6% (3)	0.406 (0.110-1.501)	52.2% (12)	27.9% (29)	**2.821 (1.120-7.105)**
	**No**	50.9% (27)	79.7% (59)		57% (45)	85.4% (41)		65.5% (72)	82.4% (14)		47.8% (11)	72.1% (75)	
**Walker**	**Yes**	37.7% (20)	33.8% (25)	1.188 (0.569-2.478)	39.2% (31)	29.2% (14)	1.568 (0.727-3.384)	36.4% (40)	29.4% (5)	0.729 (0.240-2.220)	26.1% (6)	37.5% (39)	0.588 (0.214-1.168)
	**No**	62.3% (33)	66.2% (49)		60.8% (48)	70.8% (34)		63.6% (70)	70.6% (12)		73.9% (17)	62.5% (65)	
**Crutches-cane**	**Yes**	20,8% (11)	58,1% (43)	**0,189 (0.084-0.424)**	35.4% (28)	54.2% (26)	**0.465 (0.224-0.965)**	43.6% (48)	35.3% (6)	0.705 (0.243-2.041)	17.4% (4)	48.1% (50)	**0,227 (0.072-0.714)**
	**No**	79.2% (42)	41.9% (31)		64.6% (51)	45.8% (22)		56.4% (62)	64.7% (11)		82.6% (19)	51.9% (54)	
**Internal caregiver**	**Yes**	22.6% (12)	16.7% (12)	1.463 (0.599-3.575)	17.7% (14)	21.7% (10)	0.775 (0.313-1.922)	20.9% (23)	6.7% (1)	0.270 (0.034-2.163)	39.1% (9)	14.7% (15)	**3.729 (1.371-10.143)**
	**No**	77.4% (41)	83.3% (60)		82.3% (65)	78.3% (36)		79.1% (87)	93.3% (14)		60.9% (14)	85.3% (87)	
**Private caregiver**	**Yes**	39.6% (21)	33.8% (24)	1.285 (0.614-2.688)	41.8% (33)	26.7% (12)	1.973 (0.888-4.381)	39.8% (43)	12.5% (2)	**0.216 (0.047-0.998)**	52.2% (12)	32.7% (33)	2.248 (0.898-5.628)
	**No**	60.4% (32)	66.2% (47)		58.2% (46)	73.3% (33)		60.2% (65)	87.5% (14)		47.8% (11)	67.3% (68)	
**Public caregiver**	**Yes**	50.9% (27)	58.9% (43)	0.725 (0.355-1.477)	58.2% (46)	51.1% (24)	1.336 (0.646-2.761)	57.3% (63)	43.8% (7)	0.580 (0.202-1.671)	60.9% (14)	54.4% (56)	1.306 (0.519-3.285)
	**No**	49.1% (26)	41.1% (30)		41.8% (33)	48.9% (23)		42.7% (47)	56.3% (9)		39.1% (9)	45.6% (47)	
**Economic level**	**<11.200 euros/year**	50.9% (27)	44.6% (33)	1.290 (0.636-2.617)	51.9% (41)	39.6% (19)	1.647 (0.796-3.409)	48.2% (53)	41.2% (7)	0.753 (0.267-2.121)	52.2% (12)	46.2% (48)	1.273 (0.515-3.144)
	**>11.200 euros/year**	49.1% (26)	55.4% (41)		48.1% (38)	60.4% (29)		51.8% (57)	58.8% (10)		47.8% (11)	53.8% (56)	
**Educational level**	**No education**	82.7% (43)	91.9% (68)	0.422 (0.140-1.268)	84.6% (66)	93.8% (45)	0.367 (0.098-1.374)	88.1% (96)	88.2% (15)	1.016 (0.208-4.9569	82.6% (19)	89.3% (92)	0.568 (0.163-1.975)
	**Has formal education**	17.3% (9)	8.1% (6)		15.4% (12)	6.3% (3)		11.9% (13)	11.8% (2)		17.4% (4)	10.7% (11)	
**Five-year survival**	**Alive**	20.8% (11)	51.4% (38)	**0.248 (0.111-0.555)**	26.6% (21)	58.3% (28)	**0.259 (0.121-0.553)**	32.7% (36)	76.5% (13)	**0.149 (0.045-0.491)**	8.7% (2)	45.2% (47)	**0.116 (0.026-0.518)**
	**Deceased**	79.2% (42)	48.6% (36)		73.4% (58)	41.7% (20)		67.3% (74)	23.5% (4)		91.3% (21)	54.8% (57)	

Table S2: Group 2: Barthel Index activities that require mobility and balance to perform and are relevant to personal independence. Association between these activities and sociodemographic factors, community living, need for mobility aids and/or home care assistants. Impact of dependence on group 2 activities on survival. 1: lives independently (alone or with a partner) - lives with other people (children, people who are neither children nor partners)

Barthel Index: activities associated with mobility as a factor of personal independence										
Social factor	Activity	Dressing - Undressing Level		Statistical test	Grooming Level		Statistical test	Bathing Level		Statistical test
	Level	Dependent	Needs help or independent	Odds ratio (Confidence interval)	Dependent	Independent	Odds ratio (Confidence interval)	Dependent	Independent	Odds ratio (Confidence interval)
**Housing situation**	**Lives independently**	20% (8)	44.8% (39)	**0.308 (0.127-0.744)**	22.6% (16)	47% (31)	**0.401 (0.190-0.848)**	34.8% (32)	42.9% (15)	0.711 (0.321-1.575)
	**lives with other people**	80% (32)	55.2% (48)		73.8% (45)	53% (35)		65.2% (60)	57.1% (20)	
**Leaving home**	**Homebound**	52.5% (21)	29.9% (26)	0.471 (0.218-1.019)	42.6% (26)	28.8% (19)	0.544 (0.261-1.136)	37% (34)	31.4% (11)	0.782 (0.341-1.793)
	**He leaves his house**	47.5% (19)	70.1% (61)		57.4% (35)	71.2% (47)		63% (58)	68.6% (24)	
**Wheelchair**	**Yes**	52.5% (21)	23% (20)	**3.703 (1.669-8.212)**	44.3% (27)	21.2% (14)	**2.950 (1.356-6.414)**	35.9% (33)	22.9% (8)	1.888 (0.770-4.627)
	**No**	47.5% (19)	77% (67)		55.7% (34)	78.8% (52)		64.1% (59)	77.1% (27)	
**Walker**	**Yes**	27.5% (11)	39.1% (34)	0.591 (0.261-1.338)	34.4% (21)	36.4% (24)	0.919 (0.443-1.903)	34.8% (32)	37.1% (13)	0.903 (0.402-2.026)
	**No**	72.5% (29)	60.9% (53)		65.6% (40)	63.6% (42)		65.2% (60)	62.9% (22)	
**Crutches-cane**	**Yes**	22.5% (9)	51.7% (45)	**0.271 (0.115-0.636)**	31.1% (19)	53% (35)	**0.401 (0.194-0.828)**	38% (35)	54.3% (19)	0.517 (0.235-1.136)
	**No**	77.5% (31)	48.3% (42)		68.9% (42)	47% (31)		62% (57)	45.7% (16)	
**Internal caregiver**	**Yes**	30% (12)	14.1% (12)	**2.607 (1.048-6.484)**	29.5% (18)	9.4% (6)	4.047 (1,482-11.050)	24.2% (22)	5.9% (2)	**5.101 (1.130-23.024)**
	**No**	70% (28)	85.9% (73)		70.5% (43)	90.6% (58)		75.8% (69)	94.1% (32)	
**Private caregiver**	**Yes**	50% (20)	29.8% (25)	**2.360 (1.086-5.130)**	41.7% (25)	31.3% (20)	1.571 (0.752-3.283)	44.9% (40)	14.3% (5)	**4.898 (1.740-13.785)**
	**No**	50% (20)	70.2% (59)		58.3% (35)	68.8% (44)		55.1% (49)	85.7% (30)	
**Public caregiver**	**Yes**	55% (22)	55.8% (48)	0.968 (0.455-2.057)	52.5% (32)	58.5% (38)	0.784 (0.388-1.585)	57.1% (52)	51.4% (18)	1.259 (0.576-2.753)
	**No**	45% (18)	44.2% (38)		47.5% (29)	41.5% (27)		42.9% (39)	48.6% (17)	
**Economic level**	**<11.200 euros/year**	52.5% (21)	44.8% (39)	1.360 (0.642-2.882)	49.2% (30)	45.5% (30)	1.161 (0.578-2.333)	47.8% (44)	45.7% (16)	1.089 (0.499-2.376)
	**>11.200 euros/year**	47.5% (19)	55.2% (48)		50.8% (31)	54.5% (36)		52.2% (48)	54.3% (19)	
**Educational level**	**No education**	79.5% (31)	92% (80)	0.339 (0.113-1.014)	83.3% (50)	92.4% (61)	0.410 (0.131-1.277)	84.6% (17)	97.1% (34)	0.162 (0.020-1.280)
	**Has formal education**	20.5% (8)	8% (7)		16.7% (10)	7.6% (5)		15.4% (14)	2.9% (1)	
**Five-year survival**	**Alive**	12.5% (5)	50.6% (44)	**0.140 (0.050-0.390)**	26.2% (16)	50% (33)	**0.356 (0.168-0.751)**	30.4% (28)	60% (21)	**0.292 (0.130-0.655)**
	**Deceased**	87.5% (35)	49.4% (43)		73.8% (45)	50% (33)		69.6% (64)	40% (14)	

Table S3: Group 3: Barthel Index activities in which mobility and balance are not essential to their performance and are relevant to personal independence. Association between these activities and sociodemographic factors, community living, need for mobility aids and/or home caregivers. Impact of dependence on group 3 activities on survival. 1: lives independently (alone or with a partner) - lives with other people (children, people who are neither children nor partners)

**Barthel Index: activities associated with personal care**										
**Social factor**	**Activity**	**Feeding**		**Statistical test**	**Bladder**		**Statistical test**	**Bowels**		**Statistical test**
		**Level**			**Level**			**Level**		
	**Level**	**Dependent or needs help**	**Independent**	**Odds ratio (Confidence interval)**	**Dependent**	**Independent**	**Odds ratio (Confidence interval)**	**Dependent**	**Independent**	**Odds ratio (Confidence interval)**
**Housing situation[1]**	**Lives independently**	10.5% (4)	48.3% (43)	**0.126 (0.041-0.384)**	29.5% (13)	41% (34)	0.604 (0.277-1.320)	15.4% (4)	42.6% (43)	**0.245 (0.079-0.764**)
	**lives with other people**	89.5% (34)	51.7% (46)		70.5% (31)	59% (49)		84.6% (22)	57.4% (58)	
**Leaving home**	**Homebound**	39.5% (15)	33.7% (30)	0.780 (0.356-1.709)	47.7% (21)	28.9% (24)	**0.446 (0.209-0.951)**	46.2% (12)	32.7% (33)	0.566 (0.236-1.360)
	**He leaves his house**	60.5% (23)	66.3% (59)		52.3% (23)	71.1% (59)		53.8% (14)	67.3% (68)	
**Wheelchair**	**Yes**	39.5% (15)	29.2% (26)	1.580 (0.714-3.499)	43.2% (19)	26.5% (22)	2.107 (0.975-4.553)	50% (13)	27.7% (28)	**2.607 (1.077-6.308)**
	**No**	60.5% (23)	70.8% (63)		56.8% (25)	73.5% (61)		50% (13)	72.3% (73)	
**Walker**	**Yes**	28.9% (11)	38.2% (34)	0.659 (0.290-1.498)	29.5% (13)	38.6% (32)	0.668 (0.305-1.464)	23.1% (6)	38.6% (39)	0.477 (0.176-1.292)
	**No**	71.1% (27)	61.8% (55)		70.5% (31)	61.4% (51)		76.9% (20)	61.4% (62)	
**Crutches-cane**	**Yes**	28.9% (11)	48.3% (43)	**0.436 (0.193-0.985)**	27.3% (12)	50.6% (42)	**0.366 (0.166-0.807)**	30.8% (8)	45.55 (46)	0.531 (0.212-1.334)
	**No**	71.1% (27)	51.7% (46)		72.7% (32)	49.4% (41)		69.2% (18)	54.5% (55)	
**Internal caregiver**	**Yes**	34.2% (13)	12.6% (11)	**3.593 (1.430-9.028)**	27.3% (12)	14.8% (12)	2.156 (0.874-5.321)	26.9% (7)	17.2% (17)	1.777 (0.646-4.888)
	**No**	65.8% (25)	87.4% (76)		72.7% (32)	85.2% (69)		73.1% (19)	82.9% (82)	
**Private caregiver**	**Yes**	57.9% (22)	26.7% (23)	**3.766 (1.689-8.396)**	50% (22)	28.8% (23)	**2.478 (1.154-5.320)**	53.8% (14)	31.6% (31)	**2.522 (1.045-6.084)**
	**No**	42.1% (16)	73.3% (63)		50% (22)	71.3% (57)		46.2% (12)	68.4% (67)	
**Public caregiver**	**Yes**	47.4% (18)	59.1% (52)	0.623 (0.290-1.340)	54.5% (24)	56.1% (46)	0.939 (0.450-1.961)	53.8% (14)	56% (56)	0.917 (0.385-2.180)
	**No**	52.6% (20)	40.9% (36)		45.5% (20)	43.9% (36)		46.2% (12)	44% (44)	
**Economic level**	**<11.200 euros/year**	55.3% (21)	43.8% (39)	1.584 (0.737-3.401)	50% (22)	45.8% (38)	1.184 (0.570-2.462)	38.5% (10)	49.5% (50)	0.638 (0.264-1.539)
	**>11.200 euros/year**	44.7% (17)	56.2% (50)		50% (22)	54.2% (45)		61.5% (16)	50.5% (51	
**Educational level**	**No education**	81.1% (30)	91% (81)	0.423 (0.141-1.268)	86% (37)	89.2% (74)	0.750 (0.248-2.266)	92.3% (249	87% (87)	1.793 (0.378-8.497)
	**Has formal education**	18.9% (7)	9% (8)		14% (6)	10.8% (9)		7.7% (2)	13% (13)	
**Five-year survival**	**Alive**	21.1% (8)	46.1% (41)	**0.312 (0.129-0.756)**	27.3% (12)	44.6% (37)	0.466 (0.211-1.029)	19.2% (5)	43.6% (44)	**0.308 (0.108-0.883)**
	**Deceased**	78.9% (30)	53.9% (48)		72.7% (32)	55.4% (46)		80.8% (21)	56.4% (57)	
