# Supplementary material for: Association between the different basic activities of daily living on the Barthel index and community living, use of mobility aids, and the survival at 5 years
Source: Front Public Health. 2026 Jun 4;14:1825340. doi: 10.3389/fpubh.2026.1825340 (PMC13277337; doi:10.3389/fpubh.2026.1825340)
Supplement: Supplementary file 3 [file Supplementary_File_3.pdf]

Appendix 3: Data behind Figures 1, 2, and 3.

Figure 1: Analysis of the capacity for discrimination against Barthel Index activities in which mobility is a necessary factor for their performance as a test to assess survival (group 1). Comparison with the Barthel Index.

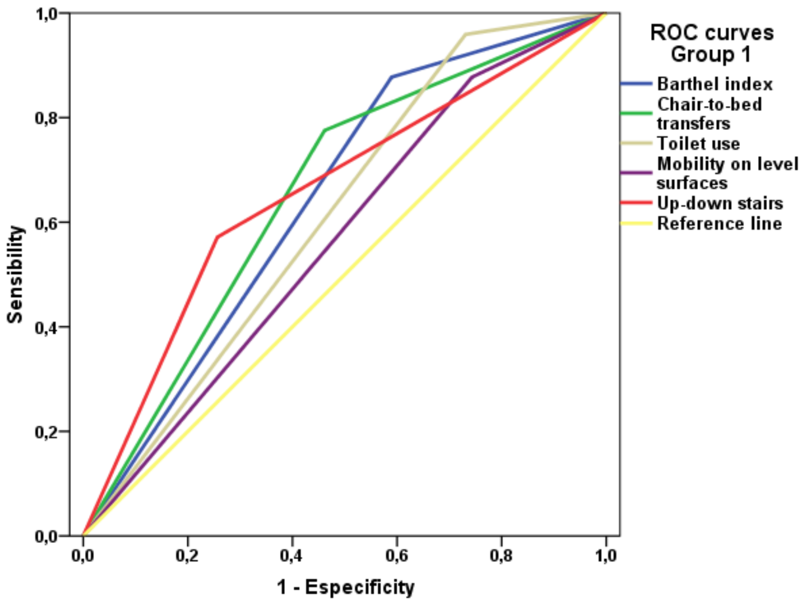

ROC curves Barthel index and mobility activities

Variables	Area	Std. Error	Sig.	95% CI Lower	95% CI Upper
Barthel index	0.644	0.049	0.006	0.548	0.740
Chair-to-bed transfers	0.657	0.049	0.003	0.561	0.753
Toilet use	0.614	0.049	0.031	0.517	0.711
Mobility on level surfaces	0.567	0.051	0.205	0.466	0.668
Up-down stairs	0.658	0.051	0.051	0.558	0.757

Curva	Área ROC	EE (DeLong)	IC(95%)	
1	0,6439	0,0367	0,5720	0,7158
2	0,6570	0,0414	0,5759	0,7381
3	0,6575	0,0435	0,5722	0,7428
4	0,5670	0,0343	0,4997	0,6343
5	0,6142	0,0290	0,5573	0,6711

Prueba de homogeneidad de áreas

Ji-cuadrado	gl	Valor p
4,3767	4	0,0364

Figure 2: Analysis of the discriminatory capacity of Barthel index activities in which mobility is associated but not essential factors for their performance, as a test to assess survival (group 2). Comparison with the Barthel Index.

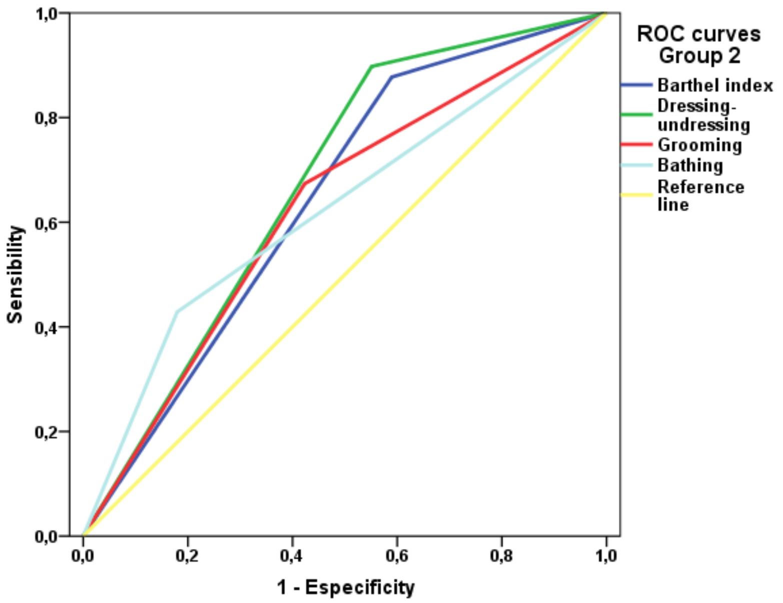

ROC curves Barthel index and personal care activities

Variables	Area	Std. Error	Sig.	95% CI Lower	95% CI Upper
Barthel index	0.644	0.049	0.006	0.548	0.740
Dressing-undressing	0.673	0.048	0.001	0.580	0.766
Grooming	0.625	0.051	0.018	0.526	0.725
Bathing	0.625	0.052	0.018	0.522	0.727

Curva	Área ROC	EE (DeLong)	IC(95%)	
1	0,6439	0,0367	0,5720	0,7158
2	0,6733	0,0358	0,6032	0,7435
3	0,6252	0,0440	0,5389	0,7115
4	0,6245	0,0419	0,5425	0,7066

Prueba de homogeneidad de áreas

Ji-cuadrado	gl	Valor p
1,0690	3	0,3012

Figure 3: Analysis of the discriminatory capacity of Barthel Index activities that do not require mobility as a necessary factor for their performance as a test to assess survival (group 3). Comparison with the Barthel Index.

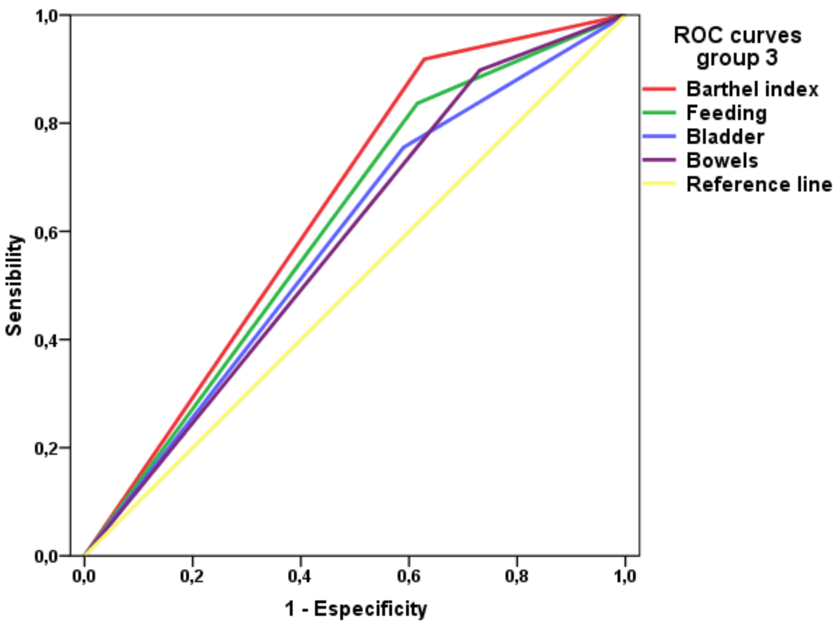

ROC curves group 3

Variables	Area	Std. Error	Asymptotic significance	95% CI	
				Lower	Upper
Barthel index	0.645	0.049	0.006	0.550	0.740
Feeding	0.611	0.050	0.036	0.512	0.709
Bladder	0.583	0.051	0.118	0.482	0.683
Bowel	0.584	0.051	0.114	0.484	0.683

Curva	Área ROC	EE (DeLong)	IC(95%)	
1	0,3893	0,0385	0,3139	0,4647
2	0,4173	0,0418	0,3354	0,4993
3	0,4164	0,0334	0,3509	0,4819
4	0,3561	0,0367	0,2842	0,4280

Prueba de homogeneidad de áreas

Ji-cuadrado	gl	Valor p
1,8421	3	0,1747
